# The consciousness of virtue: uncovering the gaps between educational specialists and the general public in their understanding of virtue in Japan

**DOI:** 10.3389/fpsyg.2023.1171247

**Published:** 2024-02-15

**Authors:** Koji Tachibana, Eisuke Nakazawa

**Affiliations:** ^1^Faculty of Humanities, Chiba University, Chiba, Japan; ^2^Pellegrino Center for Clinical Bioethics, Georgetown University Medical Center, Washington, DC, United States; ^3^Faculty of Medicine, Department of Biomedical Ethics, The University of Tokyo, Tokyo, Japan

**Keywords:** moral consciousness, Japanese society, Buddhism, Christianity, Confucianism, virtue ethics, virtue epistemology, positive psychology

## Abstract

Virtue is a normative concept that constitutes social and moral codes. The notion of virtue can be identified in both the West and the East. Since this concept was revived in academia in the 20th century after a long sinking into oblivion, contemporary professional researchers, but not the general public, may be familiar with this notion. We conducted a survey on the attitudes of educational specialists and the general public regarding the notion of virtue in Japan. Our study found that, in contrast to educational specialists, the general public were not so familiar with the notion; both had a positive image of virtue and a poor understanding of the Confucian notions of virtue; both retain Buddhist values under the term of virtue, but educational specialists tended to associate ancient Greek and Western elements with the notion of virtue. Educational specialists emphasized active, intellectual virtues, such as practical wisdom, whereas the general public emphasized passive, emotional virtues, such as gratitude. Our study showed that, the notion of virtue was understood in different ways between educational specialists and the general public in Japan. This finding has several social implications, such as academic integrity and educational policy.

## 1 Introduction

### 1.1 Brief history of virtue

Virtue is a normative concept that can be identified in both the West and the East. In the West, this concept is derived from the ancient Greek word *aretē*, which originally meant ‘excellence’ of any kind. Although this meaning was kept throughout the ancient Greek world, from Homer to Plato, Aristotle, and the Stoics, it is particularly translated as ‘virtue’ when applied to the excellence of human beings (Liddell et al., 1996, p. 238). Plato and Aristotle do not agree on what constitutes human virtue. Even the Stoics offer a diverse kind of virtues (von Arnim, 1903, sections 255–307). However, they all agree that ‘virtue’ refers to the excellence that one should acquire as a human being. This conceptual position remained the same after the influx of Christianity, when human virtues came to be understood as the four cardinal virtues in relation to the Christian God (Pieper, 1965).

In the East, specifically Far East Asia, the concept of virtue came from Confucianism. References to this concept can already be found in the Four Confucian Classics (ancient Confucian scriptures). What makes the Confucian notion of virtue different from that of the West, as is evident in Confucius’ *Analects*, is that the notion of virtue is considered the excellence to be possessed by rulers, such as kings and monarchs, rather than by every human being, including the general public (Confucius, 1998). Virtuous rulers possess a variety of useful characteristics for governing their people reliably and making their society stable.

Historically speaking, the ancient concept of ‘virtue’ was lost later in both Western and Eastern societies. It was only in the middle of the 20th century that the notion was revived in these societies. In the intellectual history of the West, Nietzsche (1874) memorably stated that it was impossible to think things seriously with the term of virtue. Such loss in the West is said to be caused by the development of modern values, such as individualism (MacIntyre, 1981), and the actual breakdown of traditional social institutions and political upheavals (Frede, 2013). In 1958, the idea of virtue was revived in academia under the form of virtue ethics, pioneered by Anscombe (1958). In the Far East, especially China, the idea of virtue lost its power in the midst of various social and political changes. However, in the same year, contemporary neo-Confucian scholars revived the Confucian idea of virtue (Mou et al., 1958). At present, ethical and epistemological studies of virtue have flourished both in Western and Eastern academia, especially in the fields of philosophy and education.

### 1.2 Prior research and a missing link

In recent years, a wide range of studies on virtue have been published that go beyond the so-called philosophical approach. They can be divided into three main categories. The first is research that focuses on the ubiquity of the idea of virtue in various cultures. This category includes research on the similarities and differences between Confucian and Greek–Christian notions of virtue (Yu, 2007), research from a broader view of virtue based on these differences (Boyd and Timpe, 2021), and research on non-Christian or non-Confucius conceptions of virtue, such as virtue in Islam and Africa (Bucar, 2018; Ogunyemi, 2021). Second, studies have sought to reveal virtue itself in collaboration with empirical sciences. This category includes studies linked to psychology and sociology (Kristjánsson, 2013; Wright et al., 2020; Fowers, 2021) and to psychiatry and neuroscience (Tachibana et al., 2017; Tachibana, 2020). Third, we can also find a more recent trend that attempts to clarify the conception of virtue in a country from its national curriculum of education. This category includes a study focused on the code of moral education in the Chinese government to clarify the structure of the character strengths in this code through a questionnaire survey (Huo et al., 2022).

Accumulation of such theoretical and empirical research will lead us to a well-known philosophical dispute about the comparison between morality and virtue: Morality is universal, non-subjective, and can be observed and even aimed at in various eras and areas, whereas virtue is either considered as also universal (Nussbaum, 1988) or, like other social conventions, are local, subjective, and can differ in different eras and different areas (MacIntyre, 1981). We will not examine this issue because it requires more philosophical study design. Still, it is worth noting that such recent research suggests ‘the lack of agreement across cultures and historical periods as to which qualities count as virtues’ (Nucci and Ilten-Gee, 2021, chap. 4). Taking this situation into consideration, we have room to investigate the consciousness of virtue in a specific culture. In particular, given that the notion of virtue has been revised in the academic context, it is worth examining to what extent the general public (GP) of the region today, being compared with educational specialists (ESs) such as professional researchers, is familiar with and understands the notion of ‘virtue’ in the culture.

However, prior research leaves the answer to this question unclear. One might argue that while there may not be specific research looking at the GP and ES differences on the notion of virtue, there must be similar research comparing the differences in some aspect of value or ideology. Still, we must first identify which aspect(s) of value or ideology has been assumed to be related to the notion of virtue in a culture. To answer this question, a survey on the consciousness of virtue is required.

The forementioned question on the consciousness of virtue is worthy of investigation when we take into account the abovementioned three points: (1) The concept of virtue was once lost in both the West and the East, (2) it was academically revived by researchers in the mid-twentieth century, and (3) the notion of virtue can differ by culture and historical period. Such a trajectory of the notion of virtue suggested by these historical facts opens the following two possibilities:

1. Unlike ESs, the notion of virtue may be unfamiliar to the GP.
2. The GP may understand the notion in a different way to ESs.

These two possibilities suggest that the GP may use the concept of virtue differently from ESs.

### 1.3 Aim of this study

This study examined to what extent the GP and ESs in Japan share or do not share their understanding of the notion of virtue with each other. In the absence of previous research, this study must be exploratory. Making this study a milestone, future studies will provide more definitive answers to this question.

### 1.4 Setting concrete research questions to accomplish the aim

To achieve the aim of this study, we focus on the possible changes in people’s conceptual understanding of the word ‘virtue’ in the field of study, Japan. As mentioned in Section 1.2, the conceptual understanding of the word ‘virtue’ in both the West and the East has been influenced by changes in values and specific political events in society. Japan is no exception. In particular, the Japanese concept of virtue, influenced by both Eastern (Confucianism) and Western (ancient Greek and Christianity) traditions, is formed through a mix of both [Tachibana, n.d., forthcoming (a)]. Based on this historical understanding of the Japanese notion of ‘virtue’, we set up six categories listed below, which we can assume to have some influence on the changes in the conceptual understanding of the word ‘virtue’ (the list of virtues in six categories is presented in Table 1).

**Table 1:** List of virtues in six categories.

Category					
1	2	3	4	5	6
**Western moral virtues**	**Western epistemic virtues**	**Psychological Virtues**	**Imperial Japanese moral and epistemic virtues**	**Confucian virtues**	**Post-War Japanese moral and epistemic virtues**
Courage (勇気)	Memory (記憶力)	Creativity (独創性)	Loyalty (忠君)	Benevolence (仁)	Independence, autonomy, freedom and responsibility (自主、自律、自由と責任)
Temperance (節制)	Eyesight (視力)	Curiosity (好奇心·興味)	Filial devotion (孝行)	Loyalty (忠)	Temperance (節度、節制)
Prudence (思慮深さ)	Hearing ability (聴力)	Open-mindedness (判断)	Fidelity (信義)	Deference (恕)	Ambition (向上心、個性の伸長)
Justice (正義)	Olfaction (嗅覚)	Love of learning (向学心)	Non-impoliteness (無礼でないこと)	Trustworthiness (信)	Hope, courage, self-denial, strong will (希望と勇気、克己と強い意志)
Piety (敬虔さ)	Sense of taste (味覚)	Perspective (見通し)	Studying (修学)	Ritual propriety (礼)	Searching for truth, creation (真理の探究、創造)
Hope (希望)	Tactility (触覚)	Bravery (勇敢)	Serving the public (剬共奉仕)	Wisdom (知)	Compassion, gratitude (思いやり、感謝)
Love (愛)	Reasoning (推論能力)	Persistence (勤勉)	Patriotism (愛国心)	Righteousness (義)	Politeness (礼儀)
Generosity (気前よさ·物惜しみのなさ)	Autonomy (自律性)	Integrity (誠実性)	Self-sacrifice (自己犠牲)	Culture (文)	Friendship, trust (友情、信頼)
Pride (志の高さ)	Understanding (理解力)	Vitality (熱意)	Local patriotism (郷土愛)	Constant mean (中庸)	Mutual-understanding, broad-mindedness (相互理解、寛容)
Love for fame (名誉愛)	Humility (謙遜)	Love (愛する力·愛される力)		Filial piety (孝)	Spirit of law observance, spirit of public morality (遵法精神、剬徳心)
Mild-temperedness (温和さ)	Non-discretion (非独断性)	Kindness (親切)		Fraternal respect (弟(悌))	Fairness, equity, social justice (剬正、剬平、社会正義)
Sociality (社交性)	Non-gullibility (騙されにくさ)	Social Intelligence (社会的知能)		Respectfulness (恭)	Social participation, public spirit (社会参画、剬共の精神)
Honesty (正直さ)	Non-egoism (非自己中心性)	Leadership (リーダーシップ)		Reverence (敬)	Laboring (勤労)
Wit (機知)	Non-self-complacency (自己満足で終わらないこと)	Forgiveness and mercy (寛大)		Deference (譲)	Family love, repletion of home life (家族愛、家庭生活の充実)
Sense of shame (羞恥心)	Non-cruelness (冷徹でないこと)	Humility and modesty (謙虚)		Humbleness (謙)	Better school life, repletion of group life (よりよい学校生活、集団生活の充実)
Legitimacy (合法)	Responsibility (責任)	Prudence (思慮深さ·慎重)		Humility (孫(遜))	Respect for local tradition and culture, love for hometown (郷土の伝統と文化の尊重、郷土を愛する態度)
Equity (剬平さ)	Carefulness (慎重さ)	Self-regulation (自己コントロール)		Earnest (勤)	Respect for national tradition and culture, love for nation (我が国の伝統と文化の尊重、国を愛する態度)
Chastity (純潔·貞操)	Negligence (怠慢でないこと)	Appreciation of beauty (審美心)		Uprightness (直)	International understanding, international contribution (国際理解、国際貢献)
Abstinence (禁欲)	Non-overconfidence (過信しないこと)	Gratitude (感謝)		Stubbornness (諒)	Dignity of life (生命の尊さ)
Humility (謙遜)	Persistence (粘り強さ)	Hope (希望·楽観性)		Genial (良)	Nature conservation (自然愛護)
Effort (努力)	Enthusiasm (熱意)	Humor (ユーモア·遊戯心)		Carefulness (慎)	Inspiration, awe-inspiring (感動、畏敬の念)
Non-envy (嫉妬のなさ)	Attentiveness (注意力)	Spirituality (精神性)		Courage (勇)	Joy of living better (よりよく生きる喜び)
Trust (信頼)	Sensitiveness (敏感さ)			Elegancy (斯文)	
Gratitude (感謝)	Self-discipline (自己鍛錬)			Fate (命)		Compassion (共感)	Reflection (反省)			Arbiter of person (天)	
				Way (道)	

In contemporary Japanese academia, the notion of virtue is said to be revived by introducing Western contemporary virtue ethics (The Japanese Society for Ethics, 1994; Tachibana, 2021). Since then, this term has come to be referred to by professional philosophers through Japanese translations of Western literature on virtue ethics. As mentioned in Section 1.1, Western contemporary virtue ethics is the theory that has revived the notion of virtue in ancient Greek philosophy and Christianity. Therefore, we can expect that such Western moral virtues also came to be shared by researchers in the fields of education and the philosophy of education who were involved in the study of moral education in Japan (**Category 1: Western moral virtues**).

Following the same trajectory, Japanese translations of Western virtue epistemology began publication in the 2010s and have been increasing thereafter. Since the 1980s, virtue epistemology has studied the human excellence as the subject of epistemic agency and, accordingly, provided lists of epistemic virtues (Zagzebski, 1996; Sosa, 2007; Greco, 2010). Introducing such Western virtue epistemology, the term ‘virtue’ in Japanese has come to be used to refer to both the ethical and epistemic aspects of virtue among ESs (Tachibana, 2021). Therefore, we can also expect that such Western epistemic virtues have a certain influence on the Japanese understanding of the notion of virtue (**Category 2: Western epistemic virtues**).

Similar to Western academia mentioned in Section 1.2, some Japanese professional researchers, including educational psychologists and scientific philosophers, started to pay attention to the notion of virtue that can be found as ‘character strengths and virtues’ in the field of positive psychology (Otake et al., 2005; Shimai, 2006). Since it remains uncertain to what extent such a psychology-laden notion of virtue has influenced Japanese understanding of the notion of virtue, we allocated one category to the list of virtues presented in positive psychology (**Category 3: Psychological Virtues**).

However, we must not overlook a certain image that seems to be widely shared by Japanese society, namely, the image that the Japanese notion of virtue has a strong connection with the pre-war, highly feudalistic Confucian view of virtue, which was reinforced by a commitment to militarism and totalitarianism (The Japanese Society for Ethics, 1994). Since this understanding of Japanese notion of virtue was crystalized in the Imperial Rescript on Education, we allocated one category to such Imperial Japanese moral and epistemic virtues (**Category 4: Imperial Japanese moral and epistemic virtues**).

Historically speaking, such a pre-war, highly feudalistic Confucian notion of virtue is not the original image of the Japanese notion of virtue. In the 5th century A.D., the Confucian concept of virtue entered Japan along with Buddhism. However, Buddhism became the predominant religion in Japan. In this respect, Japan belongs to a Buddhist rather than a Confucian culture. More than 1,200 years later, in the late 17th century, the Tokugawa shogunate readopted the Confucian notion of virtue, based on the doctrines of Zhu Xi. Therefore, the so-called traditional Confucian notion of virtue can also have a certain influence on the Japanese understanding of the notion of virtue (**Category 5: Confucian virtues**).

After World War II, in the face of resistance to the pre-war, extremely feudalistic Confucian virtues, the Japanese government newly established the Basic Law on Education, which aimed at belittling Confucian colorations and introducing the Western notion of virtue in 1947 and, accordingly, has enforced a new moral education system as a sort of hybrid of the ancient Greek concept of virtues and Confucian virtues since 1958 [Tachibana, n.d., forthcoming (a, b)]. The new system adopts the Western notion of virtue and sets the aim of moral education to be character formation as human beings but also includes some less feudalistic Confucian virtues that should be taught as components of such formation. Since the The Japanese Ministry of Education, Culture, Sports, Science and Technology (MEXT) (2017) published the list of virtues to be taught at schools, we allocated one category to them (**Category 6: Post-War Japanese moral and epistemic virtues**).

These six categories of the notion of virtue probably have influenced the Japanese understanding of virtue. Taking the reception history of virtues in Japan into consideration, the aim of this study can be structured with the following three concrete research questions concerning the possible gap between the GP and ESs regarding their understanding of the notion of virtue:

1. **On familiarity with the notion of virtue:** To what extent is the word ‘virtue’, which philosophers have advocated reviving, familiar to the GP? To what extent does the frequency with which the word is seen, heard, or used by the GP differ from that of ESs? (Answered through Result 3.1 and Discussion 4.1.)
2. **On the image of the notion of virtue:** Japanese society experienced a major backlash against the Confucian idea of virtue (and its education) twice: at the time of Japan’s opening to the West in the mid-19th century and after World War II. Hence, what kind of impression does people have of the word ‘virtue’? (Answered through Result 3.2 and Discussion 4.2.) What kinds of notions do they associate with this word? (Answered through Result 3.3 and Discussion 4.3.)
3. **On lost and important virtues:** As in the West, the idea of ‘virtue’ has been temporarily forgotten in Japan. Which virtues have become difficult for contemporary Japanese people to understand? (Answered through Result 3.4 and Discussion 4.4.) Which virtues do they think are important? (Answered through Result 3.5 and Discussion 4.5.)

By answering these structured questions, this study clarified people’s consciousness of virtue, namely, to what extent the GP and ESs have gaps in their understanding of the notion of virtue. (It should be noted that this study is not conducted to uncover the conceptual relationship between categories, because such an interesting study would require a different study design.)

## 2 Methods

### 2.1 Participants

A survey was conducted on the GP and ESs residing in Japan. For the survey of the GP, a research consulting company was commissioned to conduct the survey. Among the panelists registered with the company, the GP other than ESs were equally allocated in their 20s to 60s (five age groups) and in both sexes. They were recruited until 1,000 responses were received. (Therefore, the mean age and variance do not fit the type of data.) Those who expressed their willingness to participate in the survey were asked to respond to an online questionnaire.

Regarding the survey of ESs, to efficiently recruit professionals in the fields of philosophy and education, we asked the following ten academic societies related to those fields to inform their members via e-mail:

1. Japanese Society for Ethics,
2. Philosophical Association of Japan,
3. Japan Association for Bioethics,
4. Japanese Educational Research Association,
5. Philosophy of Education Society of Japan,
6. Society for High School Education of Ethics and Modern Society in Tokyo,
7. Space Humanities and Social Sciences in Japan Society for Aeronautical and Space Sciences,
8. Chiba City Board of Education Secretariat,
9. Chiba City Institute for Future City Planning, and
10. Slack Philosophy Online Seminar.

Those who agreed to participate in the survey were asked to respond to an online questionnaire.

**Figure 1:**
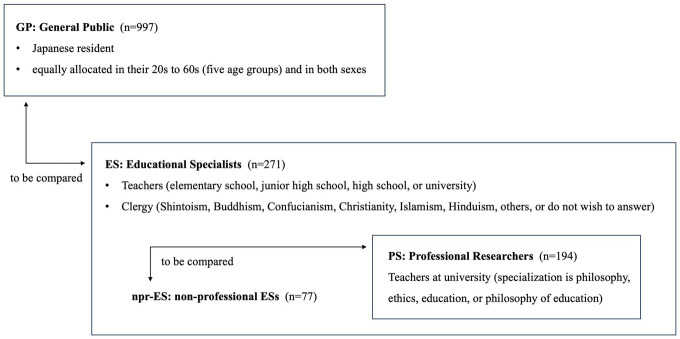
Relationships among study participants.

ESs were defined as those who selected one of the following 14 categories as their profession: six categories of teachers [(1) elementary school, (2) junior high school, (3) high school, (4) university (specialization is philosophy, including ethics), (5) university (specialization is education, including philosophy of education) and (6) university (specialization is other than philosophy or education)] and eight categories of clergy [(7) Shinto, (8) Buddhist, (9) Confucian, (10) Christian, (11) Islam, (12) Hinduism, (13) others, and (14) do not wish to answer]. Clergypersons are classified into ESs for two reasons. First, they have rich knowledge of the general concept of morality, which includes moral virtues. Second, looking back at Japanese history, they have taken the role of teachers in local communities. They had schools called *Terakoya* and gave lectures on moral topics at such schools and during their ritual services. (At present, we can find Shinto, Buddhist, Confucian, Christian, Islamic, and Hindu schools in Japan.) Of these 14 occupational categories of ESs, (4) and (5) were designated as ‘professional researchers (PRs)’ concerning the notion of virtue in any sense, whereas the 12 other occupational categories were designated as ‘non-professional ESs (npr-ESs).’ The relationships among GP, ES, PR, and npr-ES are shown in Figure 1.

### 2.2 Procedure

The survey on the GP was conducted from February 1 to February 4, 2022. The survey targeting ESs was conducted from February 28 to March 25, 2022.

For both surveys on the GP and ESs, the survey items were identical as follows (Supplementary material 1). As basic attributes, the respondents were asked about their final education (7 categories), occupation (24 categories, including 6 categories for teachers and 8 categories for clergy), age (for the professional survey, age group), gender (4 categories), and religion (9 categories) (Q1–5).

Twelve questions were then established to answer the three questions embodied above in Section 1.4 (Q6–17).

First, to examine the first question, which was on the familiarity of the notion of ‘virtue’, we investigated the experience of exposure to the word ‘virtue’ (Q6–7). Here, respondents were asked about the frequency with which they see or hear the word ‘virtue’ in their daily lives and the frequency with which they use the word ‘virtue’ in their daily lives by using a five-point Likert scale ranging from ‘not at all’ to ‘almost every day’.

Next, to examine the second question, which was on the image of ‘virtue,’ we investigated the impression of the word and what one associates with it (Q8–9). The respondents were asked about their impressions of the word ‘virtue’ by using a five-point Likert scale ranging from ‘very unfavourable impression’ to ‘very favourable impression.’ To examine associations with the word ‘virtue,’ the respondents were asked to select first, second, and third place from the 41 virtue-related words/phrases that we collated in advance (see also the last paragraph of Section 2.3.1).

Finally, to examine the third question, which was on lost and important virtues, we investigated the virtues whose meaning they could hardly understand (Q10) and the virtues they considered important (Q11–16). For the 129 virtues included in the list of virtues, the respondents were asked to provide multiple responses regarding virtues whose meanings they could hardly understand. In doing so, the option ‘No words whose meaning I do not understand’ was added. The 129 virtues were then divided into six categories, and the respondents were asked to select the first to fifth most important virtues in each category. The question was not asked in the form of ‘choose important virtues that are associated with the word “virtue” ’ because the respondents’ answers could be biased by their own (mis-)understanding of ‘virtue’. Conversely, by asking the question in the form of ‘what is important for a person *qua* person’, it becomes possible to clarify what character traits they assume important as virtues, even if respondents have rarely seen or heard the word ‘virtue’ or have a negative impression of the word.

### 2.3 Measure

#### 2.3.1 How to create a virtue list

In the questionnaire, the list of virtues from Categories 1–6 was presented in Japanese (Supplementary material 1). The list in each category was developed as follows. Category 1, ‘Western Moral Virtues’, lists the virtues mentioned in the complete works of Plato and Aristotle as ancient Greek philosophers, respectively, and those of Christianity, which can be summarized as theological and cardinal virtues, are listed together (Pieper, 1965; Barnes, 1984; Cooper, 1997). As there were various Japanese translations of these works, we followed the most popular Japanese translations of the virtues in these works (Plato, 1974–1978; Aristotle, 2015–2016). Among these virtues, it should be noted that ‘prudence (or practical wisdom)’ is listed as a moral virtue. Although Aristotle categorizes it into intellectual virtue (*dianoētike aretē*), it is a virtue related to so-called ethical practice and not to so-called intellectual/epistemic activity in contemporary virtue epistemology. As the term ‘intellectual virtues’ was used in the context of contemporary virtue epistemology in this article, practical wisdom was classified into moral virtues.

Category 2, ‘Western Epistemic Virtues’, organizes and lists the virtues discussed in the survey papers of contemporary virtue epistemology (Greco, 2002; Kvanvig, 2010; Battaly, 2015; Turri and Alfano, 2017). However, since contemporary virtue epistemology has not yet been introduced well in Japan (Tachibana, 2021), there was no fixed Japanese translation for each virtue yet. Therefore, in this study, the closest Japanese translations used in the other categories were adopted in this category.

Category 3, ‘Psychological Virtues’, lists the items proposed by positive psychology (Peterson and Seligman, 2004). For the Japanese translation, we adapted the expressions used by previous studies (Otake et al., 2005; Shimai, 2006; Aoki, 2008; Aoki et al., 2022).

Category 4, ‘Imperial Japanese Moral and Epistemic Virtues’, lists the virtues in the ‘Imperial Rescript on Education’ (1890), which served as the foundation of moral education in the Empire of Japan until 1945 and can be sorted out into nine virtues (Takahashi, 2017). The item name ‘moral and epistemic’ is used here because it includes the virtue of ‘studying’. However, given that other virtues are moral virtues, it might be possible to lump them together under moral virtues. Considering that the original language of this category was Japanese, a Japanese translation was not necessary for the participants in the study. The closest English translations used in the other categories were adopted in writing this article.

Category 5, ‘Confucian Virtues’, lists virtues found in the *Analects* (Kimura, 1975). Given that the original words are in Chinese, a Japanese translation was not necessary. Chinese characters can have more than one meaning. Although many Japanese people learn how to read Chinese poems in high school, they may not fully understand Chinese sentences. Nevertheless, Japanese people can understand most Chinese characters used in such sentences because the Japanese language also uses many Chinese characters. Therefore, Japanese people can also understand the several meanings that a single Chinese character has. This implies two things. First, Japanese people do not need Japanese translation of most Chinese characters. Second, even if Japanese people translate a Chinese character into Japanese, they will adopt the same character as the Japanese translation. For example, unless we make an explanatory translation, the Japanese translation of the Chinese character ‘天’ is ‘天’. Therefore, Japanese translations were not presented to the participants. English translations presented in this article are based on Confucius (1938, 1998).

Category 6, ‘post-war Japanese Moral and Epistemic Virtues’, lists the virtues that should be taught in current Japanese moral education. Here, 22 virtues are listed for upper elementary and junior high school students based on the ‘Commentary on the Courses of Study for Elementary Schools’ (originally announced in 2009) published by the The Japanese Ministry of Education, Culture, Sports, Science and Technology (MEXT) (2017). Japanese translations were not required for this category, either, as the original language was Japanese. The closest English translations used in the other categories were used in this article.

We do not claim that the 129 virtues are exhaustively sufficient to capture the very notion of virtue in itself because the concept of virtue is both philosophically and historically complicated. However, we believe that the virtues listed in Table 1 are comprehensive enough to cover the range of the notion of virtue that the six categories represent in the history of Japan.

The list of words associated with virtue was also presented to the participants in Japanese (Supplementary material 1). The list was developed as follows. First, it was confirmed that the list must be developed to study which term contemporary Japanese people, who are expected to understand the historical and cultural transition of Japan, can associate with virtue. Next, the authors assume that people would not be able to write down sufficient terms if they were requested to do so in a free-description format, because such an association can be subconscious if the notion of virtue is not used so often in their daily lives (this assumption was later confirmed by this study). Thus, possible items need to be presented in a multiple-choice format. Among the 41 items, 39 items, except for “Others” and “Nothing,” are selected in advance by the authors, who have grown up with Japanese culture: born in Japan as Japanese, educated through the Japanese school curriculum, and speak Japanese as their first language. In the process of selecting the items, the authors referred to several texts on the subjects of *Civics* (including *Ethics*) and *Japanese History*, which were authorized by the MEXT for use in Japanese high schools (Sasayama et al., 2021; Takeuchi et al., 2021). Although the authors believe the list provides possible terms in as much good balance as possible, there remains a possibility that it may contain some bias from the authors.

#### 2.3.2 Analysis

We counted the respondents’ basic attributes of Final Education, Occupation, Age, Sex, and Religion, and determined the percentage of the total number for each attribute. On the experience of exposure to and the impression of the word ‘virtue,’ the percentages of each option in the group were determined for the GP, ES, PR, and npr-ES groups. To compare the distributions of GP and ES groups, and that of PR and npr-ES groups, Fisher’s exact probability test was performed. For the words associated with the word ‘virtue’, the words ranked first were included in the tally, and the percentage of each word that ranked first was determined. To compare GP and ES as well as PR and npr-ES groups for each of the associating words, we determined the difference in proportions and effect size, and performed Fisher’s exact probability test, then adjusted for multiplicity using Holm’s method. For virtues whose meanings were not understood, the percentage selected for each virtue was obtained for each group. After determining the difference between the corresponding proportions of GP and ES, and npr-ES and PR, and determining the effect size, Fisher’s exact probability test was performed with adjustment for multiplicity using Holm’s method. For the virtues that were considered important, the ratio of the first place mentioned for each category was obtained for each category. In addition, for the virtues that were considered important, we tested the difference in proportions for each virtue for the combinations of the GP and ES as well as PR and npr-ES groups. We performed Fisher’s exact probability and further adjusted for multiplicity for each question using Holm’s method. All statistical analysis was conducted using SAS software, version 9.4 (SAS Institute Inc., Cary, NC, USA), where *p* < 0.05 was considered significant (SAS, 2022).

## 3 Results

### 3.1 Attributes of the target population and the frequency of exposure to the word ‘virtue’ in daily life

Table 2 shows the basic attributes. The GP was surveyed through registered panelists of the research consulting company, and responses were obtained from 1,000 respondents (8,364 were asked to complete the survey, with a response rate of 12.0%). Three persons who were operationally defined as ESs were excluded, and 997 persons were included in the analysis as members of the GP. Regarding age, the respondents were equally sampled from their 20s to 60s, and the ratio of males to females was set at 1:1. Multiple responses were allowed for religion, with 449 (45.0%) selecting no particular religion, 339 (34.0%) selecting Buddhism, and 56 (5.6%) selecting Shintoism in descending order (excluding those who did not wish to answer).

**Table 2:** Basic attributes of the participants.

		GP		ES					
			(%)	Total	(%)	PR	(%)	npr-ES	(%)
Total number		997		271		194		77	
Final Education	Secondary school	20	2.0%	0	0.0%	0	0.0%	0	0.0%
	High school or passed the high school equivalency examination	344	34.5%	0	0.0%	0	0.0%	0	0.0%
	Technical school	152	15.2%	1	0.4%	0	0.0%	1	1.3%
	Junior college	87	8.7%	0	0.0%	0	0.0%	0	0.0%
	University (undergraduate)	358	35.9%	19	7.0%	2	1.0%	17	22.1%
	University (master’s degree)	30	3.0%	59	21.8%	38	19.6%	21	27.3%
	University (Ph.D.)	6	0.6%	192	70.8%	154	79.4%	38	49.4%
Occupation	Student (Technical school, Junior College)	4	0.4%	0	0.0%	0	0.0%	0	0.0%
	Student (Undergraduate, Graduate)	27	2.7%	0	0.0%	0	0.0%	0	0.0%
	Part-time worker	173	17.4%	0	0.0%	0	0.0%	0	0.0%
	Company employee, public servant (includes non-regular worker)	472	47.3%	0	0.0%	0	0.0%	0	0.0%
	Entrepreneur, a board member	10	1.0%	0	0.0%	0	0.0%	0	0.0%
	Independent professions	43	4.3%	0	0.0%	0	0.0%	0	0.0%
	Without occupation	180	18.1%	0	0.0%	0	0.0%	0	0.0%
	Teacher (primary school) *	0	0.0%	5	1.8%	0	0.0%	5	6.5%
	Teacher (secondary school) *	0	0.0%	9	3.3%	0	0.0%	9	11.7%
	Teacher (High school) *	0	0.0%	13	4.8%	0	0.0%	13	16.9%
	Teacher (University) (Specialty is philosophy, including ethics) *	0	0.0%	107	39.5%	107	55.2%	0	0.0%
	Teacher (University) (Specialty is education, including philosophy of education) *	0	0.0%	87	32.1%	87	44.8%	0	0.0%
	Teacher (University) (Specialty is other than philosophy or education) *	0	0.0%	45	16.6%	0	0.0%	45	58.4%
	Medical workers	43	4.3%	0	0.0%	0	0.0%	0	0.0%
	Professional business workers (lawyer, certificated public accountant, etc.)	5	0.5%	0	0.0%	0	0.0%	0	0.0%
	Clergy (Shintoism)	0	0.0%	2	0.7%	0	0.0%	2	2.6%
	Clergy (Buddhism)	0	0.0%	2	0.7%	0	0.0%	2	2.6%
	Clergy (Confucianism)	0	0.0%	0	0.0%	0	0.0%	0	0.0%
	Clergy (Christianity)	0	0.0%	0	0.0%	0	0.0%	0	0.0%
	Clergy (Islamism)	0	0.0%	0	0.0%	0	0.0%	0	0.0%
	Clergy (Hinduism)	0	0.0%	0	0.0%	0	0.0%	0	0.0%
	Clergy (Others)	0	0.0%	1	0.4%	0	0.0%	1	1.3%
	Clergy (do not wish to answer)	0	0.0%	0	0.0%	0	0.0%	0	0.0%
	Others	40	4.0%	0	0.0%	0	0.0%	0	0.0%
Age	Teens (high school student or younger)	-	-	0	0.0%	0	0.0%	0	0.0%
	Teens (student at technical school, junior college, university or worker)	-	-	0	0.0%	0	0.0%	0	0.0%
	Twenties	198	19.9%	7	2.6%	2	1.0%	5	6.5%
	Thirties	200	20.1%	56	20.7%	44	22.7%	12	15.6%
	Forties	199	20.0%	90	33.2%	66	34.0%	24	31.2%
	Fifties	200	20.1%	65	24.0%	43	22.2%	22	28.6%
	Sixties	200	20.1%	51	18.8%	37	19.1%	14	18.2%
	Seventies	-	-	2	0.7%	2	1.0%	0	0.0%
	Eighties	-	-	0	0.0%	0	0.0%	0	0.0%
	Nineties and more	-	-	0	0.0%	0	0.0%	0	0.0%
Sex	Male	498	49.9%	187	69.0%	141	72.7%	46	59.7%
	Female	499	50.1%	76	28.0%	46	23.7%	30	39.0%
	Others	-	-	1	0.4%	1	0.5%	0	0.0%
	Do not wish to answer	-	-	7	2.6%	6	3.1%	1	1.3%
Religion	Shintoism	56	5.6%	25	9.2%	17	8.8%	8	10.4%
	Buddhism	339	34.0%	80	29.5%	56	28.9%	24	31.2%
	Confucianism	0	0.0%	2	0.7%	2	1.0%	0	0.0%
	Christianity	18	1.8%	13	4.8%	10	5.2%	3	3.9%
	Islamism	2	0.2%	0	0.0%	0	0.0%	0	0.0%
	Hinduism	0	0.0%	0	0.0%	0	0.0%	0	0.0%
	Atheism	449	45.0%	139	51.3%	98	50.5%	41	53.2%
	Others	43	4.3%	16	5.9%	13	6.7%	3	3.9%
	Do not wish to answer	141	14.1%	17	6.3%	14	7.2%	3	3.9%

Percentages are calculated for each item. The age of the GPs was set by the survey company, sampling evenly from the 20s to the 60s. GP, general public; ES, educational specialists; PS, professional researchers; npr-ESs, non-professional educational specialists. * ‘teacher’ includes regular, non-regular, and retired teachers.

**Figure 2:**
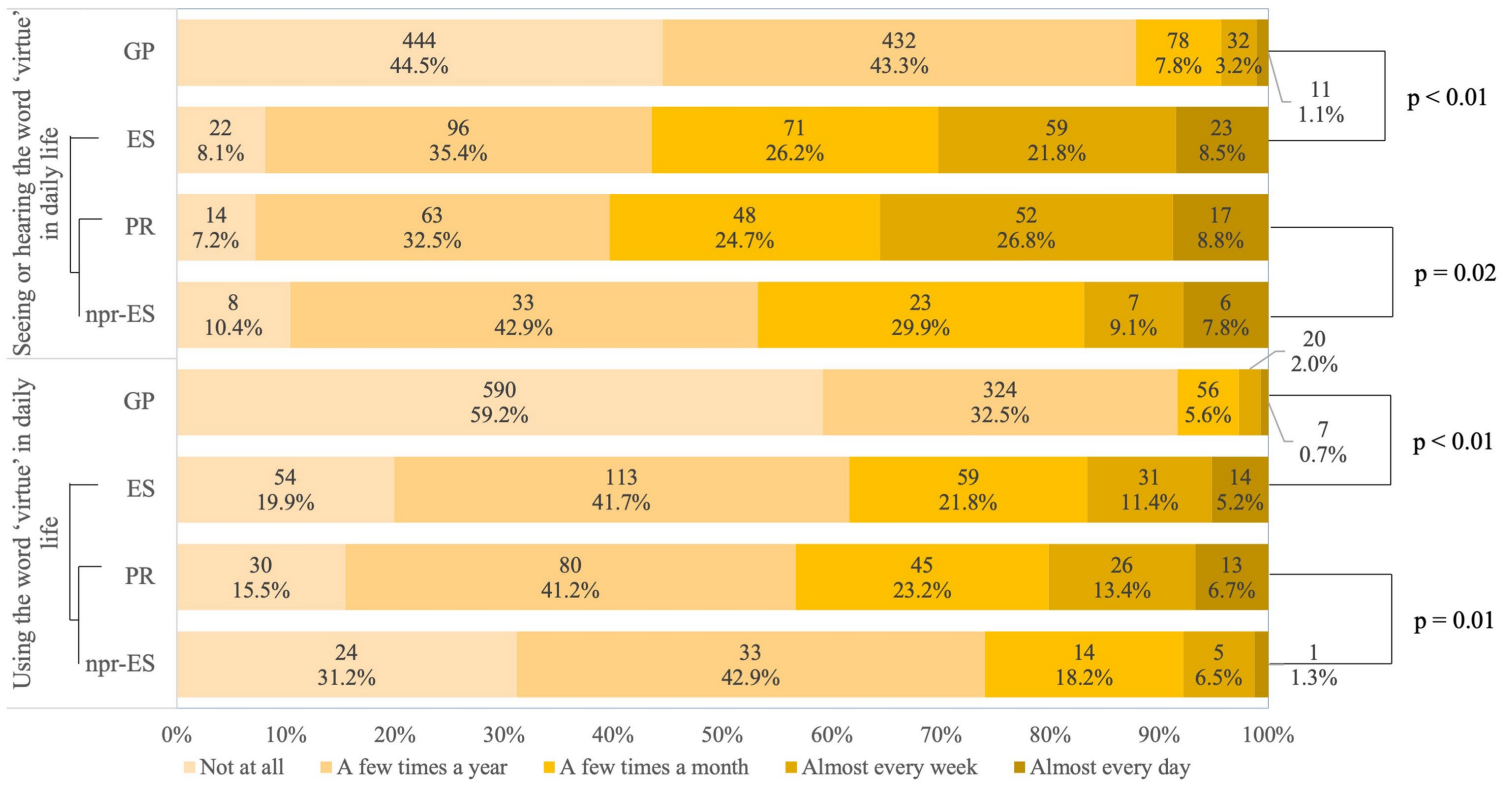
Experience of Exposure to the Word “Virtue’. The *p*-values represent the results of Fisher’s exact probability test comparing GP to ES and PR to npr-ES. Fisher’s exact probability test was adopted because the six scales are treated as qualitative variables, not ordinal variables. GP, general public; ES, educational specialists; PS, professional researchers; npr-ES, non-professional educational specialists.

As for seeing and hearing the word ‘virtue’ in daily life, a higher number of the GP (444; 44.5%) chose ‘not at all’, followed by ‘a few times a year’ (432; 43.3%). Similarly, the most frequent use of the word ‘virtue’ in daily life was ‘not at all’ (590 respondents; 59.2%), followed by ‘a few times a year’ (324 respondents; 32.5%) (Figure 2).

Of the 383 respondents to the e-mail-based survey on expecting ESs, 115 were excluded from the survey because they did not satisfy the conditions of ESs (e.g., not working in education). With three clergies from the company-based survey on the GP added, 271 respondents were classified as ESs. Among these, 194 and 77 respondents were classified into PRs and npr-ESs, respectively. As for religion, similar to the GP, the respondents selected no particular religion (139 respondents, 51.3%), Buddhism (80 respondents, 29.5%) or Shintoism (25 respondents, 9.2%), in descending order.

As for seeing and hearing the word ‘virtue’ in daily life, ‘not at all’ was selected the least by 22 respondents of the ES (8.1%), whereas ‘a few times a year’ was selected by 96 respondents (35.4%), and 82 (30.3%) respondents answered frequently (‘almost every week’ or ‘almost every day’; for the GP, this percentage was 4.3%). The most frequent use of the word ‘virtue’ in daily life was ‘a few times a year’ by 113 respondents (41.6%). Furthermore, 45 respondents (16.6%) used the word ‘virtue’ frequently, but the percentage increased to 20.1% when limited to PRs (39 respondents). Fisher’s exact probability test for differences in distribution between the GP and ESs showed significant differences both in “seeing and hearing the word ‘virtue’ in daily life” and in “using the word ‘virtue’ in daily life” (*p* < 0.01). Similarly, these differences were observed between PR and npr-ES groups (*p* = 0.02, *p* = 0.01) (Figure 2).

### 3.2 Impression of the word virtue

**Figure 3:**
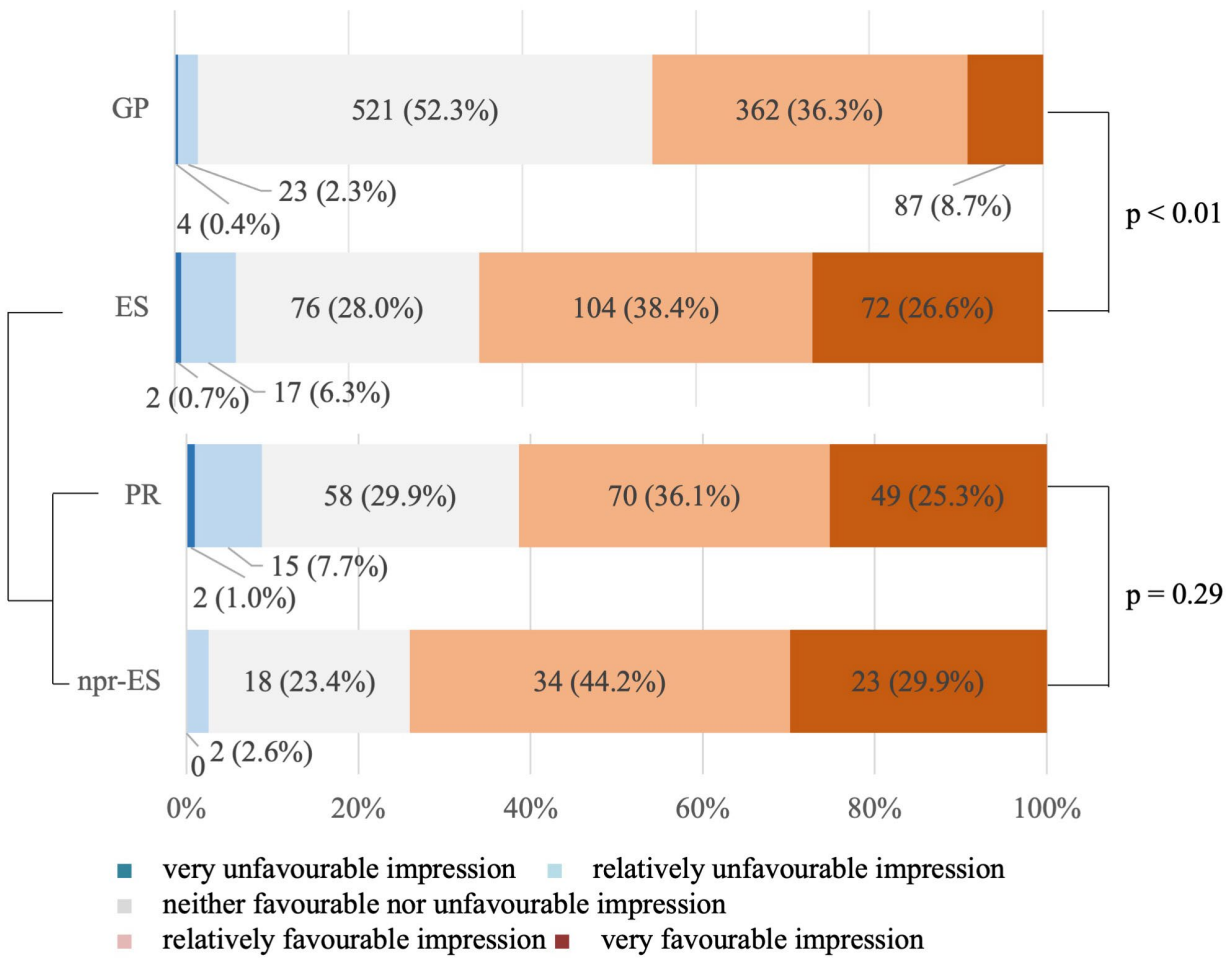
Impression of the word ‘virtue’. The *p*-values represent the results of Fisher’s exact probability test comparing GP to ES and PR to npr-ES. Fisher’s exact probability test was adopted because the five scales are treated as qualitative variables, not ordinal variables. GP, general public; ES, educational specialists; PS, professional researchers; npr-ES, non-professional educational specialists.

Figure 3 shows the results concerning the impression of the word ‘virtue’. When ‘very unfavourable impression’ and ‘relatively unfavourable impression’ were defined as the negative group, ‘relatively favourable impression’ and ‘very favourable impression’ as the positive group, and ‘neither favourable nor unfavourable impression’ as the neutral group, the positive group significantly outnumbered the negative group. For the GP, the negative group was 2.7%, whereas the positive group was 45.0%. For the ESs, the negative group was 7.0%, whereas the positive group was 65.0%. The neutral group was smaller in the ESs (28.0%) than in the GP (52.3%) (Fisher’s exact probability test comparing neutral impression and others showed *p* < 0.01), and the positive or negative impressions were more clearly depicted in the ESs than in the GP (*p* < 0.01). However, this significant difference was not detected in the comparison between PR and npr-ES (*p* = 0.29).

### 3.3 Words associated with virtue

**Figure 4:**
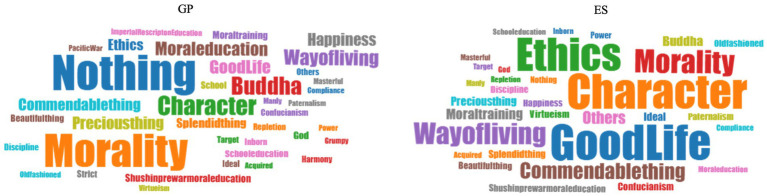
Word cloud of the words associated with virtue. The words associated with the word virtue were visualized in a word cloud for each GP and ES. The size of each character is proportional to its relative frequency of occurrence. They are created by fanbright@clah (https://lab.Fanbright.jp/wordcloud/). GP, general public; ES, educational specialists.

Table 3 summarizes the words associated with the word ‘virtue’. The most common response was ‘nothing’ (18.2% of GP), followed by ‘morality’ (16.9%), ‘character’ (8.3%), ‘Buddha’ (7.6%) and ‘way of living’ (5.9%). As for ESs, the most frequently associated words were ‘good life’ (15.9%), ‘character’ (15.5%), ‘ethics’ (14.8%), ‘morality’ (9.6%) and ‘way of living’ (8.5%), in descending order. One notable difference between the GP and ESs was the associations with ‘good life’. It was high among ESs (15.9%) and low among the GP (4.8%). Similarly, ‘ethics,’ and ‘character’ were associated more with ESs than the GP. In contrast, ‘moral education’ was the word that the GP associated with more than ESs, and more of the GP than ESs said they had no association with it. Words that evoke pre-war Japanese militaristic education and generally contained negative connotations, such as ‘World War II’, ‘Pacific War’, ‘militarism’, ‘Imperial Rescript on Education’ and ‘*Shushin*’, were rarely selected as associations by either the GP or ESs (0–1.4%). Comparing npr-ES and PR, there was no significant difference concerning the words associated with the word ‘virtue’. (Words associated with virtue in the GP and ESs were also represented in the word cloud to facilitate intuitive understanding (Figure 4).)

**Table 3:** Words associated with virtue.

	GP		ES											
		(%)	Total	(%)	Difference in proportion (GP-ES)	Effect size	*p*-value	PR	(%)	npr-ES	(%)	Difference in proportion (npr-ES-PR)	Effect size	*p*-value
Happiness (幸福)	42	4.20%	3	1.10%	3.11%	0.20		2	1.00%	1	1.30%	0.27%	0.02	
Good Life (善き生)	48	4.80%	43	15.90%	−11.05%	−0.38	**	27	13.90%	16	20.80%	6.86%	0.18	
Precious thing (大切なもの)	41	4.10%	6	2.20%	1.90%	0.11		6	3.10%	0	0.00%	−3.09%	−0.35	
Splendid thing (素晴らしいもの)	26	2.60%	4	1.50%	1.13%	0.08		2	1.00%	2	2.60%	1.57%	0.12	
Beautiful thing (美しいもの)	10	1.00%	3	1.10%	−0.10%	−0.01		1	0.50%	2	2.60%	2.08%	0.18	
Commendable thing (立派なもの)	37	3.70%	16	5.90%	−2.19%	−0.10		11	5.70%	5	6.50%	0.82%	0.03	
Repletion (充実)	6	0.60%	1	0.40%	0.23%	0.03		1	0.50%	0	0.00%	−0.52%	−0.14	
Way of living (生き方)	59	5.90%	23	8.50%	−2.57%	−0.10		17	8.80%	6	7.80%	−0.97%	−0.04	
Discipline (秩序)	7	0.70%	3	1.10%	−0.40%	−0.04		3	1.50%	0	0.00%	−1.55%	−0.25	
Harmony (調和)	5	0.50%	0	0.00%	0.50%	0.14		0	0.00%	0	0.00%	0.00%	0.00	
God (神)	14	1.40%	1	0.40%	1.04%	0.12		0	0.00%	1	1.30%	1.30%	0.23	
Buddha (仏)	76	7.60%	7	2.60%	5.04%	0.24		5	2.60%	2	2.60%	0.02%	0.00	
Ethics (倫理)	36	3.60%	40	14.80%	−11.15%	−0.41	**	31	16.00%	9	11.70%	−4.29%	−0.12	
Target (目標)	6	0.60%	1	0.40%	0.23%	0.03		1	0.50%	0	0.00%	−0.52%	−0.14	
World War II (第二次世界大戦)	0	0.00%	0	0.00%	0.00%	0.00		0	0.00%	0	0.00%	0.00%	0.00	
Pacific War (太平洋戦争)	1	0.10%	0	0.00%	0.10%	0.06		0	0.00%	0	0.00%	0.00%	0.00	
Militarism (軍国主義)	0	0.00%	0	0.00%	0.00%	0.00		0	0.00%	0	0.00%	0.00%	0.00	
Imperial Rescript on Education (教育勅語)	1	0.10%	0	0.00%	0.10%	0.06		0	0.00%	0	0.00%	0.00%	0.00	
Shushin/prewar moral education (修身)	14	1.40%	3	1.10%	0.30%	0.03		2	1.00%	1	1.30%	0.27%	0.02	
Paternalism (パターナリズム(押しつけ))	1	0.10%	3	1.10%	−1.01%	−0.15		3	1.50%	0	0.00%	−1.55%	−0.25	
Masterful (偉そう)	4	0.40%	1	0.40%	0.03%	0.01		0	0.00%	1	1.30%	1.30%	0.23	
Strict (堅苦しいもの)	9	0.90%	0	0.00%	0.90%	0.19		0	0.00%	0	0.00%	0.00%	0.00	
Grumpy (気難しいもの)	2	0.20%	0	0.00%	0.20%	0.09		0	0.00%	0	0.00%	0.00%	0.00	
Old-fashioned (古くさいもの)	3	0.30%	3	1.10%	−0.81%	−0.10		3	1.50%	0	0.00%	−1.55%	−0.25	
Morality (道徳)	168	16.90%	26	9.60%	7.26%	0.22		17	8.80%	9	11.70%	2.93%	0.10	
Virtue education (徳育)	12	1.20%	8	3.00%	−1.75%	−0.13		4	2.10%	4	5.20%	3.13%	0.17	
Moral education (道徳教育)	49	4.90%	1	0.40%	4.55%	0.33	††	0	0.00%	1	1.30%	1.30%	0.23	
Virtue-ism (徳目主義)	4	0.40%	4	1.50%	−1.07%	−0.12		4	2.10%	0	0.00%	−2.06%	−0.29	
Character (人柄)	83	8.30%	42	15.50%	−7.17%	−0.22	*	32	16.50%	10	13.00%	−3.51%	−0.10	
Confucianism (儒教)	5	0.50%	4	1.50%	−0.97%	−0.10		1	0.50%	3	3.90%	3.38%	0.25	
Ideal (理想的)	5	0.50%	5	1.80%	−1.34%	−0.13		4	2.10%	1	1.30%	−0.76%	−0.06	
School (学校)	9	0.90%	0	0.00%	0.90%	0.19		0	0.00%	0	0.00%	0.00%	0.00	
School education (学校教育)	10	1.00%	1	0.40%	0.63%	0.08		0	0.00%	1	1.30%	1.30%	0.23	
Manly (男っぽい)	2	0.20%	1	0.40%	−0.17%	−0.03		1	0.50%	0	0.00%	−0.52%	−0.14	
Womanly (女っぽい)	0	0.00%	0	0.00%	0.00%	0.00		0	0.00%	0	0.00%	0.00%	0.00	
Compliance (コンプライアンス(法令遵守))	3	0.30%	1	0.40%	−0.07%	−0.01		1	0.50%	0	0.00%	−0.52%	−0.14	
Power (力)	3	0.30%	2	0.70%	−0.44%	−0.06		2	1.00%	0	0.00%	−1.03%	−0.20	
Inborn (先天的(生まれつき))	5	0.50%	1	0.40%	0.13%	0.02		0	0.00%	1	1.30%	1.30%	0.23	
Acquired (後天的)	3	0.30%	1	0.40%	−0.07%	−0.01		0	0.00%	1	1.30%	1.30%	0.23	
Others (その他)	7	0.70%	11	4.10%	−3.36%	−0.24	**	11	5.70%	0	0.00%	−5.67%	−0.48	
Nothing (連想するものはない)	181	18.20%	2	0.70%	17.42%	0.71	**	2	1.00%	0	0.00%	−1.03%	−0.20	

We examined 39 theoretically selected candidate words associated with the word virtue. Fisher’s exact probability tests were performed on the difference between the GP and ES ratios, and between the PR and npr-ES ratios, with further multiplicity adjustment using Holm’s method. Although respondents were asked to select up to 3rd place, only 1st place was used in the analysis. The reason is that the strength of preference between the 2nd and 3rd place is unknown, and we thought that only the 1st place would better reflect the subject’s preference in the analysis. Fisher’s exact probability tests were performed on the difference between the GP and ES ratios, and between the PR and npr-ES ratios, with further multiplicity adjustment using Holm’s method. When the ratio was significantly greater for ES than for the GP and for PR than for npr-ES, * denotes *p* < 0.05, and ** denotes *p* < 0.01. When the ratio was significantly smaller for ES than for the GP and for PR than for npr-ES, † indicates *p* < 0.05, and †† indicates *p* < 0.01. GP, general public; ES, educational specialists; PS, professional researchers; npr-ES, non-professional educational specialists.

### 3.4 Virtues that are difficult to understand

To ascertain the degree of recognition of each virtue, Table 4 shows the top 10 most difficult-to-understand virtues. Both the GP and ESs found ‘elegancy’ and ‘stubbornness’ as Confucian virtues to be the most difficult to understand. Regarding the differences between the GP and ESs, ‘constant mean,’ ‘piety’, and ‘wit’ as Western moral virtues were difficult for most of the GP to understand (*p* < 0.01), but ESs understood the meaning of these virtues better than the GP. Although we listed ‘constant mean’ as a Confucian virtue, the word is also a key concept of Western moral virtues (see Section 4.4). Many of the ESs also answered that they did not understand Confucian virtues (eight of the ten virtues belong to Category 5). Furthermore, a larger percentage of ESs than the GP responded that they did not understand the meaning of ‘elegancy’, ‘fraternal respect’, ‘humility’, ‘stubbornness’, and ‘culture’ as Confucian virtues (*p* < 0.01). No significant differences were observed between PR and npr-ES groups with respect to these differences in understanding.

**Table 4:** Virtues that are difficult to understand.

Rank	GP		ES		Gap between GP and ES				Gap between npr-ES and PR			
	Virtue list	Percentage	Virtue list	Percentage	Virtue list	Percentage difference (GP-ES)	Effect size	*p*-value	Virtue list	Percentage difference (npr-ES-PR)	Effect size	*p*-value
1	Elegancy (斯文)	57.5%	Elegancy (斯文)	82.3%	Constant mean (中庸)	36.5%	0.99	††	Respectfulness (恭)	16.2%	0.39	
2	Stubborn (諒)	41.1%	Stubborn (諒)	60.5%	Piety (敬虔さ)	28.7%	0.73	††	Piety (敬虔さ)	10.4%	0.35	
3	Constant mean (中庸)	40.2%	Respectfulness (恭)	34.7%	Wit (機知)	26.5%	0.69	††	Appreciation of beauty (審美心)	10.2%	0.32	
4	Piety (敬虔さ)	36.8%	Humility (孫(遜))	31.4%	Elegancy (斯文)	−24.8%	−0.55	**	Stubborn (諒)	9.8%	0.20	
5	Wit (機知)	33.9%	Deference (恕)	22.5%	Fraternal respect (弟 (悌))	−23.5%	−0.58	**	Fraternal respect (弟 (悌))	−8.5%	−0.18	
6	Respectful (恭)	29.2%	Fraternal respect (弟 (悌))	19.6%	Humility (孫(遜))	−19.7%	−0.49	**	Deference (恕)	6.7%	0.16	
7	Appreciation of beauty (審美心)	28.7%	Love for fame (名誉愛)	18.5%	Stubborn (諒)	−19.4%	−0.39	**	Elegancy (斯文)	6.6%	0.18	
8	Love for fame (名誉愛)	27.7%	Culture (文)	16.6%	Appreciation of beauty (審美心)	19.1%	0.50	††	Constant mean (中庸)	5.7%	0.28	
9	Deference (恕)	24.7%	Social Intelligence (社会的知能)	12.9%	Culture (文)	−11.8%	−0.40	**	Humility (孫(遜))	−5.7%	−0.12	
10	No words whose meaning I do not understand	16.3%	Deference (譲)	10.7%	Spirit of law observance, spirit of public morality (遵法精神、剬徳心)	11.6%	0.44	††	Arbiter of person (天)	−5.6%	−0.21	

Study participants selected words that were difficult to understand for 129 virtues as multiple-choice options. The virtues ranked 1–10 in each group and the percentage of those who chose them are shown; for the divergence between the GP and the ESs, the case when the percentage of those who could hardly understand the meaning of that virtue in the GP exceeds that in ESs is represented by a positive sign, and the opposite is represented by a negative sign. Similarly, for the discrepancy between PRs and npr-ESs, a positive sign was used when the percentage of those who could hardly understand the meaning of that virtue in the npr-ES exceeded that in the PRs, and the opposite was represented by a negative sign. Fisher’s exact probability tests were performed on the difference between the GP and ES ratios, and between the PR and npr-ES ratios, with further multiplicity adjustment using Holm’s method. When the ratio was significantly greater for ES than for the GP and for PR than for npr-ES, * denotes *p* < 0.05, and ** denotes *p* < 0.01. When the ratio was significantly smaller for ES than for the GP and for PR than for npr-ES, † indicates *p* < 0.05, and †† indicates *p* < 0.01. GP, general public; ES, educational specialists; PS, professional researchers; npr-ES, non-professional educational specialists.

### 3.5 Virtues to be considered important

Table 5 (complete table is provided as Supplementary material 2) organizes the virtues that are considered important. For each set of virtues from category 1 to category 6, the virtues considered important by the GP and ESs were organized. Then, *P*-tests were performed to test the virtues for differences in proportions. The same procedure was conducted for the PRs and npr-ESs as a breakdown of the ESs.

**Table 5:** Virtues considered important.

Category	Ranking	Virtue	GP		ES								Total points
					Total		*p*-value	PR		npr-ES		*p*-value	
1	1	Gratitude (感謝)	264	26.5%	25	9.2%	††	12	6.2%	13	16.9%		36
	2	Love (愛)	143	14.3%	49	18.1%		33	17.0%	16	20.8%		32
	3	Honesty (正直さ)	136	13.6%	27	10.0%		21	10.8%	6	7.8%		24
	4	Trust (信頼)	95	9.5%	20	7.4%		11	5.7%	9	11.7%		17
	5	Prudence (思慮深さ)	47	4.7%	31	11.4%	**	21	10.8%	10	13.0%		16
		Hope (希望)	20	2.0%	19	7.0%	**	15	7.7%	4	5.2%		9
		Compassion (共感)	7	0.7%	20	7.4%	**	15	7.7%	5	6.5%		8
		Wit (機知)	0	0.0%	4	1.5%	*	4	2.1%	0	0.0%		2
2	1	Autonomy (自律性)	92	9.2%	69	25.5%	**	43	22.2%	26	33.8%		35
	2	Responsibility (責任)	201	20.2%	27	10.0%	††	15	7.7%	12	15.6%		30
	3	Not applicable	109	10.9%	29	10.7%		26	13.4%	3	3.9%		22
	4	Understanding (理解力)	78	7.8%	18	6.6%		12	6.2%	6	7.8%		14
	5	Reflection (反省)	34	3.4%	23	8.5%	*	20	10.3%	3	3.9%		12
		Non-discretion (非独断性)	5	0.5%	10	3.7%	**	7	3.6%	3	3.9%		4
3	1	Gratitude (感謝)	278	27.9%	26	9.6%	††	13	6.7%	13	16.9%		38
	2	Integrity (誠実性)	123	12.3%	68	25.1%	**	46	23.7%	22	28.6%		37
	3	Love (愛する力·愛される力)	96	9.6%	24	8.9%		17	8.8%	7	9.1%		19
	4	Not applicable	80	8.0%	20	7.4%		18	9.3%	2	2.6%		15
	5	Prudence (思慮深さ·慎重)	34	3.4%	25	9.2%	**	20	10.3%	5	6.5%		13
		Hope (希望·楽観性)	21	2.1%	18	6.6%	**	14	7.2%	4	5.2%		9
4	1	Fidelity (信義)	202	20.3%	73	26.9%		52	26.8%	21	27.3%		47
	2	Not applicable	219	22.0%	67	24.7%		55	28.4%	12	15.6%		47
	3	Non-impoliteness (無礼でないこと)	277	27.8%	47	17.3%	††	35	18.0%	12	15.6%		45
	4	Filial devotion (孝行)	148	14.8%	18	6.6%	††	6	3.1%	12	15.6%	††	21
	5	Studying (修学)	47	4.7%	32	11.8%	**	23	11.9%	9	11.7%		17
		Serving the public (剬共奉仕)	29	2.9%	20	7.4%	*	12	6.2%	8	10.4%		10
5	1	Fate (命)	311	31.2%	30	11.1%	††	19	9.8%	11	14.3%		42
	2	Not applicable	201	20.2%	42	15.5%		31	16.0%	11	14.3%		36
	3	Benevolence (仁)	37	3.7%	43	15.9%	**	28	14.4%	15	19.5%		20
	4	Ritual propriety (礼)	122	12.2%	13	4.8%	††	6	3.1%	7	9.1%		17
	5	Constant mean (中庸)	11	1.1%	35	12.9%	**	30	15.5%	5	6.5%		14
		Wisdom (知)	26	2.6%	20	7.4%	*	16	8.2%	4	5.2%		10
		Deference (恕)	3	0.3%	13	4.8%	**	10	5.2%	3	3.9%		5
6	1	Compassion, gratitude (思いやり、感謝)	310	31.1%	40	14.8%	††	21	10.8%	19	24.7%		46
	2	Dignity of life (生命の尊さ)	180	18.1%	39	14.4%		26	13.4%	13	16.9%		33
	3	Not applicable	86	8.6%	22	8.1%		19	9.8%	3	3.9%		17
	4	Independence, autonomy, freedom and responsibility (自主、自律、自由と責任)	32	3.2%	31	11.4%	**	26	13.4%	5	6.5%		15
	5	Fairness, equity, social justice (剬正、剬平、社会正義)	28	2.8%	32	11.8%	**	27	13.9%	5	6.5%		15
		Joy of living better (よりよく生きる喜び)	37	3.7%	29	10.7%	**	17	8.8%	12	15.6%		14
		Mutual-understanding, broad-mindedness (相互理解、寛容)	28	2.8%	31	11.4%	**	24	12.4%	7	9.1%		14
		Politeness (礼儀)	88	8.8%	2	0.7%	††	2	1.0%	0	0.0%		10
		Family love, repletion of home life (家族愛、家庭生活の充実)	67	6.7%	3	1.1%	††	2	1.0%	1	1.3%		8
		Searching for truth, creation (真理の探究、創造)	4	0.4%	17	6.3%	**	14	7.2%	3	3.9%		7

For each category, the respondents were asked to select up to the fifth most important virtue. This table indicates the top five virtues (within each category) that were selected as the most important virtue (namely, the first) for respondents. For each virtue, total points were defined as the sum of the percentage of support for GP and ES (total), and the top five total points for each category are shown. For comparisons between GP and ES and PR and npr-ES, after Fisher’s exact probability test, multiplicity was adjusted by Holm’s method and *p*-values were obtained. In this table, the virtues that were not among the top five in total points, but for which statistically significant differences were observed in the comparisons, are listed by category. When the ratio was significantly greater for ES than for the GP and for PR than for npr-ES, * denotes *p* < 0.05, and ** denotes *p* < 0.01. When the ratio was significantly smaller for ES than for the GP and for PR than for npr-ES, † indicates *p* < 0.05, and †† indicates *p* < 0.01. Respondents were asked to select up to 3rd place, but only 1st place was used in the analysis. The reason was that the strength of preference between 2nd place and 3rd place was unknown, and we thought that only using 1st place for analysis would better reflect the subject’s preference. GP, general public; ES, educational specialists; PS, professional researchers; npr-ES, non-professional educational specialists.

For Category 1, in descending order, the important virtues for the GP were ‘gratitude’, ‘love’, and then ‘honesty’, whereas those for the ESs were ‘love’, ‘prudence’, and then ‘honesty’. ‘Prudence’, ‘hope’, ‘compassion’, and ‘wit’ were significantly higher in importance for the ESs than for the GP, whereas ‘gratitude’ was more important for the GP than for the ESs.

For Category 2, the important virtues for the GP were ‘responsibility,’ ‘n/a’, and then ‘autonomy,’ whereas those for the ESs were ‘autonomy,’ ‘n/a’, and then ‘responsibility’. ‘Autonomy’, ‘non-discretion’, and ‘reflection’ were significantly higher in importance for the ESs than for the GP, and ‘responsibility’ was more important for the GP than for the ESs.

For Category 3, the important virtues for the GP were ‘gratitude’, ‘integrity’, and then ‘love’, whereas those for the ESs were ‘integrity’, ‘gratitude’, and then ‘prudence’. ‘Integrity’, ‘prudence,’ and ‘hope’ were significantly higher in importance for the ESs than the GP, whereas ‘gratitude’ was more important for the GP than the ESs.

For Category 4, the important virtues for the GP were ‘non-impoliteness’, ‘n/a’, and then ‘fidelity’, whereas those for the ESs were ‘fidelity’ ‘n/a’, and then ‘non-politeness’. ‘Studying’ and ‘serving the public’ were significantly higher in importance for the ESs than for the GP, whereas ‘filial devotion’ and ‘non-politeness’ were more important for the GP than for the ESs. Moreover, ‘filial devotion’ was significantly higher in importance for the npr-ESs than the PRs. This was the significant difference that was observed between the PRs and the npr-ESs.

For Category 5, the important virtues for the GP were ‘fate’, ‘n/a’, and then ‘ritual propriety’, whereas those for the ESs were ‘benevolence,’ ‘n/a’, and then ‘constant mean’. ‘Benevolence’, ‘deference’, ‘wisdom’, and ‘constant mean’ were significantly higher in importance for the ESs than for the GP, whereas ‘ritual propriety’ and ‘fate’ were more important for the GP than for the ESs.

For Category 6, the important virtues for the GP were ‘compassion, gratitude’, ‘dignity of life,’ and then ‘politeness’ whereas those for the ESs were ‘compassion, gratitude,’ ‘dignity of life,’ and then, ‘fairness, equity, social justice’. ‘Independence, autonomy, freedom and responsibility’, ‘searching for truth, creation’, ‘mutual understanding, broad-mindedness’, ‘fairness, equity, social justice’, and ‘joy of living better’ were significantly higher in importance for the ESs than the GP, while ‘compassion, gratitude,’ ‘politeness’, and ‘family love, repletion of home life’ were more important for the GP than the ESs.

## 4 Discussion

### 4.1 Attributes of the target population and the frequency of exposure to the word ‘virtue’ in daily life

Result 3.1 with Table 2 shows that, regardless of whether they were the GP or ESs, the number of Japanese people who self-identified as Christian or Confucian were very small. This indicates that the present survey was relatively free from any specific religious context concerning the academic trend of virtue theory. The result that the highest number of respondents in both the GP and ESs were atheists is consistent with a recent survey indicating that Japanese people have gradually lost faith in religion since the 1980s (NHK Broadcasting Culture Research Institute, 2020).

Figure 2 shows a statistically significant difference between the GP and ESs in terms of how familiar they are with the word ‘virtue’, both in terms of seeing and hearing, as well as using the word. There is also a significant difference between PR and npr-ESs both in terms of seeing/hearing and using the word. Taking this statistical result into consideration, the figure suggests that the more professional participants are in the fields of philosophy or education, the more often they are exposed to the term ‘virtue’. Given this, it seems that the term ‘virtue’ appears and is used most in the context of academic research of philosophy and education. This assumption is consistent with our literature-based survey of the post-war, historical trajectory of the notion of virtue in Japan in the Introduction (Section 1.4).

### 4.2 Impression of the word ‘virtue’

Result 3.2 with Figure 3 reveals that both the GP and ESs had a positive impression of the word ‘virtue’. Very few people in both groups had a negative impression of the word, and nearly half of the GP and more than 60% of the ESs had a positive impression of the word. This is a very significant change, given the reception history of virtue in Japan mentioned in Section 1.4. However, this data itself does not say anything about the reasons why such change has occurred. Still, we can accept this data as reliable because it is consistent with Result 3.3, which revealed the words that people associate with virtue (see Section 4.3). As for the need of further research on this respect, see the limitations (Section 5.2) below.

### 4.3 Words associated with virtue

Result 3.3 with Table 3 reveals three things. First, the result shows that both groups had a positive impression of the notion of virtue but by different reasons on what constitutes a positive evaluation of ‘virtue’. Compared to the GP, the ESs have a more positive impression due to key elements of ancient Greek-based virtue ethics; the ‘good life’, ‘character’, and ‘ethics’. In this respect, it is interesting that both the GP and ESs associated ‘Buddha’ with virtue (GP, 7.6%; ESs, 2.6%). The difference between the GP and ESs is not statistically significant in this respect (*p* = 0.07). This result is harmonized with the report in the 2018 Attitudes Survey that says Buddhism is the most prevailing religion in Japan (NHK Broadcasting Culture Research Institute, 2020). However, combining this with the abovementioned significant difference that ESs tend to understand the notion of virtue in light of ancient Greek-originated values, it may be suggested that Japanese people retain Buddhist values to some extent but come to be familiar with Western moral values through acquiring educational expertise (see also Section 4.5). (It should be noted that both the GP and the ESs associate ‘virtue’ with ‘morality’. The words ‘virtue’ and ‘morality’ do not appear to have a literal relationship in English, but in Japanese, ‘morality’ is written as ‘dou-toku (way-virtue)’, which literally includes the word ‘virtue’. Therefore, the association of virtue with morality for the Japanese people might not have any significant meaning.)

Second, Result 3.3 also provides data that show few people had a negative impression of ‘virtue’, because the words/terms that remind us of pre-war Japanese militaristic education and generally contain negative nuances, such as ‘World War II’, ‘Pacific War’, ‘militarism’, ‘Imperial Rescript on Education’, and ‘*Shushin*’, were rarely selected (0–1.4%) as associated with the word ‘virtue’. This result supports the view expressed in Section 4.2 that the historically dark nuances associated with the word ‘virtue’ in Japan have been almost dispelled in the 21st century. This result is also consistent with the previous study that suggests contemporary Japanese lay-people are not so hostile to the notion of virtue (Tachibana, 2021).

Third, a clear significant difference of ‘nothing’ between the GP and ESs suggests that the notion of virtue remains ambiguous for the GP but not for ESs. To consider only participants who are serious in responding to the questions, the research company that conducted the survey to the GP and aggregated that of ESs employs an algorithm that excludes participants who finish the survey before two and a half minutes. Thus, those who are counted as choosing ‘nothing’ can be understood as having difficulty finding words that are associated with virtue. This may support an assumption that most contemporary Japanese lay-people do not know the term ‘virtue’ (The Japanese Society for Ethics, 1994, p. 150).

### 4.4 Virtues that are difficult to understand

Result 3.4 with Table 4 shows that both the GP and ESs have a poor understanding of Confucian virtues. This indicates that the word ‘virtue’ is not well connected to Confucian concepts in contemporary Japan. This estimate is consistent with the data in Table 3 that Japanese people are separated from the religious context of Confucianism as a creed.

Conversely, ‘constant mean’, ‘piety’, and ‘wit’, which were cited as the top three most significant differences in understanding between the GP and ESs, are all virtues that appear in Aristotle’s ethics. Although we listed ‘constant mean’ as a Confucian virtue, ESs did not find this word difficult to understand. Presumably, they understood or guessed it in the light of Western moral virtues, since the word also appears as a key concept in Aristotelian virtue ethics. This supports the interpretation of Table 3 in Section 4.3 that the ESs’ understanding of virtue is associated with elements derived from ancient Greek philosophy. Considering the fact that pre-war Japanese scholars had freely read Chinese classics, this could be interpreted as showing that post-war scholars in Japan have had a tendency to enhance the study of Western classics while ignoring the study of Chinese classics.

### 4.5 Virtues to be considered important

Result 3.5 with Table 5 shows two major differences between the GP and ESs in their understanding of important virtues. First, the ESs emphasized the active, intellectual virtue of ‘prudence’, whereas the GP emphasized the passive, emotional virtue of ‘gratitude’. This contrast was commonly observed between Category 1 (Western moral virtues) and Category 3 (psychological virtues). Considering that prudence is one of the most important Western moral virtues derived from Aristotle, many of the ESs were expected to understand the implications of this word. Hence, it seems that they chose this because they knew it. However, the GP did not choose it, probably because they did not know the implications. In contrast, most of the GP placed importance on ‘gratitude’ because they knew it, for the word ‘gratitude’ in Japanese also has a Buddhist coloration that is widely rooted in Japanese society through the long acceptance of Buddhism. In fact, this word means ‘a feeling of being thankful’, and ‘thankful’ in Japanese originally comes from Buddhist contexts that meant ‘seldom’ and expanded to the meaning of gratitude (Editorial Office of Shogakukan Japanese Dictionary Section, 2003, ‘gratitude’). The gap between the GP and the ESs in this respect was consistent with the contrast that we revealed in Section 4.3, where ESs associated more Greek-derived words with virtue, whereas the GP just retained Buddhist values. Furthermore, it was also consistent with the fact that, in Categories 4 and 6, the GP placed significantly more emphasis than the ESs on ‘non-impoliteness’, ‘compassion, gratitude’ and ‘politeness’, which have passive, emotional, and Buddhist colorations in Japan, whereas the ESs placed significantly more emphasis than the GP on ‘studying’, ‘independence, autonomy, freedom and responsibility’ and ‘fairness, equity, social justice’, which have active and intellectual colorations and also have strong relation to Western values in Japanese society.

The second difference, as seen in Category 5 (Confucian virtues), is concerned with a contrast in the understanding and misunderstanding of Confucian virtues. As revealed in Section 4.4, both the GP and ESs had a limited understanding of Confucian virtues. The contrast in question is, therefore, that each group chose virtues that they thought they understood the meaning and implications of. For example, the GP holds ‘fate (命)’ in extremely high esteem. ‘命’ in Japanese usually means ‘biological survival’ or ‘life’. However, ‘命’ as a Confucian virtue means ‘fate as one’s going’ or ‘one’s own mission’. It seems unlikely that post-war Japanese ordinary people highly estimate their fate or missions. Rather, it is more reasonable to assume that they probably misunderstood the meaning as an ordinary Japanese. In contrast, the ES ranked ‘benevolence (仁)’ and ‘constant mean (中庸)’ among the top three important virtues, and these two were significantly higher than the GP’s choices because it is a relatively well-known fact for ESs that ‘仁’ is the highest of the Confucian virtues and that ‘中庸’ is the key concept of both the Western and the Confucian notions of virtue. Taking these into consideration, we can estimate that such confusions happened to the GP because the similar characters were used to express different notions between Japanese and Chinese (see, Aoki et al., 2022). This misunderstanding provides a significant suggestion to our study because it may indicate that Japanese GP has forgotten the Confucian connotation of such characters.

Finally, it should be noted that ‘not applicable’ was the second most frequently chosen option by both the GP and ESs in Categories 2, 4, and 5. Among them, the result in Category 2 is particularly interesting because it deals with contemporary virtue epistemology, the value of which PRs are expected to appreciate. However, many PRs also chose ‘not applicable’ in this category: ‘not applicable’ was the second most preferred answer among the GP, ESs, and PRs. This finding can be interpreted in two ways. First, ESs (and PRs) generally but probably unconsciously agree that ‘autonomy’ is one of the most important epistemic virtues, but have not yet reached such an agreement about the other epistemic virtues. Since different participants chose different virtues as the most important epistemic virtues, the vote was split and, accordingly, ‘not applicable’ appeared as the second most preferred answer. The second possible interpretation is that a significant number of ESs (and PRs) thought that there was no important epistemic virtue. Although both interpretations are worthy of further investigation, this should be the subject of future research because it is beyond the scope of our study here.

## 5 Conclusion

### 5.1 Findings

We provide five findings concerning the question posed about the possible gaps in the understanding of ‘virtue’ between the GP and ESs.

First, with regard to the familiarity with the concept of virtue, there is a significant contrast between the GP and ESs in terms of both exposure to and use of the word ‘virtue’. ESs come into contact with this word frequently, whereas the GP does not come into contact with it very often.

Second, there is no difference in the impression of the word ‘virtue’ between the GP and ESs. Very few people in both groups had a negative impression of the word, and many of them had a positive impression of the word.

Third, there is a difference between the GP and ESs in terms of what constituted their positive impressions. Although both the GP and ESs see Buddhist elements to some extent in their positive impressions, the ESs see more ancient Greek-based virtue ethical elements in their positive impressions.

Fourth, both the GP and ESs have a poor understanding of Confucian virtues.

Fifth, there are some contrasts between the GP and ESs in their important virtues, based on the differences in their familiarity with the notion of virtue. In particular, the GP emphasizes more passive, emotional, and Buddhist values, whereas the ESs emphasizes more active, intellectual, and Western values.

As surveyed in the Introduction, this sort of study has never been conducted. Our findings concerning the gap between the GP and ESs reveal that the Japanese notion of virtue is certainly affected by the six categories we presented and that the consciousnesses of virtue that the GP and ESs entertain are the amalgam of these categories but in different proportions between them. Such influences and resulting differences constitute the gap between the GP and ESs.

These findings will have not only scientific but also social significance. We do not think that the GP must use the notion in the same way as ESs. Conversely, we believe that ESs should be cautious about the possible gap that they may have with the GP to avoid scientific and practical misfortunes for the following two reasons.

First, experimental philosophers have shown the academic harms that the ignorance of such gap can cause. They argue that professional philosophers tend to examine a concept (such as freedom of the will, intention, and autonomy) based on their intuitional understanding of the concept and construct an argument without taking into account how the GP understands and uses the concept in question. Such arguments have been criticized as self-righteous because they may not only result in an arrogant way of thinking but also provide a less feasible theory of the concept (Knobe and Nichols, 2008; Knobe and Nichols, 2014; Systma and Buckwalter, 2016). Similar problems may arise with regard to the notion of virtue if ESs neglect the gap that our study has shown between them and the GP.

Second, neglecting the gap can also cause practical harm because ESs tend to become a member of a committee for developing a new national curricula and educational policies, and also (especially in Japan) a member of an editorial board of authorized textbooks used in elementary to high schools. Virtue is a concept that describes character traits that apply to the general population rather than to particular social classes unless one adopts a very ancient Confucian view. Such a notion of virtue partly constitutes existing educational curricula and contributes to developing a new educational policy to inculcate desirable character traits. Therefore, if there is a gap between the GP and ESs on the subject of virtues, and if ESs construct a discussion or publish textbooks without noticing (or neglecting) the gap, it could not only contain errors in theoretical and empirical research on the concept of virtues, but could also be detrimental to the actual design of a education system. Therefore, it is an important effort from both theoretical and practical perspectives to be conscious and cautious about the gaps between the GP and ESs with regard to their understanding of virtue.

### 5.2 Limitations

As mentioned in the Introduction, this study was exploratory in nature. We expect that further studies will be accumulated in the future to provide a more detailed understanding of the gap between the two groups and their backgrounds.

Finally, for that future, we mention three issues that require further investigation. First, as mentioned in Section 4.2, the factors that may have caused the change in impressions of the word ‘virtue’ need to be identified. Our study has proposed a possible reason for such change. In the 70 years since the end of World War II, Japanese people have forgotten the history of the role of Confucian virtues in Japanese imperialism. Alternatively, the connotation of the word ‘virtue’ has changed from the feudal Confucian one to the Western one, namely, the realization of a good life as an individual. This reason seems probable because they are consistent with the data that when PRs and npr-ESs are compared, the latter experts tend to have a more favorable view, whereas the former tend to have a negative view. The more they understand the notion of virtue, the more they discern the historically negative aspects of the word in Japan.

Second, this study excluded schoolteachers involved in public education from the survey because, as mentioned above, the Japanese government defines the content and implications of education to a certain degree through the Courses of Study (The Japanese Ministry of Education, Culture, Sports, Science and Technology (MEXT), 2017). Given that schoolteachers carefully read these courses, they are likely to gravitate toward these implications in their responses. However, we believe that schoolteachers are at the core of moral education in Japan, and accordingly, their understanding of the notion of virtue needs to be examined in the future.

Third, this study focuses on Japan as a field where the Western and Far Eastern notions of virtue intermingle, and such intermingling makes the Japanese word ‘virtue’ complicated. However, Japan is not the only region that has such a complex meaning of virtue. What is important is to understand what the GP and ESs in each society understand by the word ‘virtue’. Further investigations on these issues will contribute to clarifying the similarities and differences in the understanding of virtue and then to promoting international and academic research on virtue.

## Data availability statement

The original contributions presented in the study are included in the article/Supplementary material, further inquiries can be directed to the corresponding author.

## Ethics statement

The studies involving humans were approved by Research Ethics Committee, Faculty of Humanities, Chiba University. The studies were conducted in accordance with the local legislation and institutional requirements. The ethics committee/institutional review board waived the requirement of written informed consent for participation from the participants or the participants’ legal guardians/next of kin because every participant responds to the survey online. Descriptions concerning informed consent are displayed to them before they start the survey. Those who only push the button of agreement can respond to the survey.

## Author contributions

KT: conceptualization and project administration. KT and EN: writing—original draft preparation and writing—review and editing. EN: statistical analysis. All authors have read and agreed to the published version of the manuscript.
