# Supplementary material for: The consciousness of virtue: uncovering the gaps between educational specialists and the general public in their understanding of virtue in Japan
Source: Front Psychol. 2024 Feb 15;14:1171247. doi: 10.3389/fpsyg.2023.1171247 (PMC10902463; doi:10.3389/fpsyg.2023.1171247)
Supplement: Supplementary file 1 [file Data_Sheet_1.pdf]

Supplementary 1. Questionnaire

[* The questionnaire was presented in Japanese. This English translation is only for the use of information as a supplement to our article.]

This questionnaire was designed to be used in research to investigate changes in values by ascertaining impressions of the modern Japanese language and includes the following information.

・Final education and religion

・Instructions about and associations with words

All survey responses will be processed and used in such a way that individuals cannot be identified. We understand that some questions may be difficult to answer, but we ask for your cooperation and understanding of the purpose of the survey. We may also ask you some questions about yourself and your family.

1. **Please provide your last level of education.**
    - Junior high school
    - High school or Certificate of achieving the proficiency level of high school graduates
    - Technical school
    - Junior college
    - University (undergraduate)
    - University (master's degree)
    - University (Ph.D.)

2. **Please indicate your occupation.**
   * 'teacher' includes regular, non-regular, and retired teachers.
    - Student (Technical school, Junior College)
    - Student (Undergraduate, Graduate)
    - Part-time worker
    - Company employee, public servant (includes non-regular workers)
    - Entrepreneur, a board member
    - Independent professions

- Without occupation
- Teacher (primary school) *
- Teacher (secondary school) *
- Teacher (high school) *
- Teacher (University) (Specialty is philosophy, including ethics) *
- Teacher (University) (Specialty is education, including philosophy of education) *
- Teacher (University) (Specialty is other than philosophy or education) *
- Medical workers
- Professional business workers (lawyer, certificated public accountant, etc.)
- Clergy (Shintoism)
- Clergy (Buddhism)
- Clergy (Confucianism)
- Clergy (Christianity)
- Clergy (Islamism)
- Clergy (Hinduism)
- Clergy (Others)
- Clergy (do not wish to answer)
- Others

3. Please indicate your age.

4. Please indicate your gender.

- Male
- Female
- Others
- Do not wish to answer

5. Please indicate your religious affiliation.

- Shintoism
- Buddhism
- Confucianism
- Christianity
- Islamism
- Hinduism
- Atheism
- Others

- Do not wish to answer

6. In your daily life, how often do you see or hear the word "virtue"?

*This is "virtue (徳)" alone, excluding idioms such as "morality (道徳)" and "meritorious deed (功徳)".

- Not at all
- A few times a year
- A few times a month
- Almost every week
- Almost every day

7. In your daily life, how often do you use the word "virtue"?

*This is "virtue (徳)" alone, excluding idioms such as "morality (道徳)" and "meritorious deed (功徳)".

- Not at all
- A few times a year
- A few times a month
- Almost every week
- Almost every day

8. What is your impression of the word "virtue"?

*This is "virtue (徳)" alone, excluding idioms such as "morality (道徳)" and "meritorious deed (功徳)".

- very unfavourable impression
- relatively unfavourable impression
- neither favourable nor unfavourable impression
- relatively favourable impression
- very favourable impression

9. What do you associate with the word "virtue"? Please choose in order the top three things you associate more strongly with the word. If not on the list, please choose "other (   )" and fill in the blank (must be a word).

(1) Happiness (幸福)
(2) Good Life (善き生)

(3) Precious thing (大切なもの)

(4) Splendid thing (素晴らしいもの)

(5) Beautiful thing (美しいもの)

(6) Commendable thing (立派なもの)

(7) Repletion (充実)

(8) Way of living (生き方)

(9) Discipline (秩序)

(10)Harmony (調和)

(11)God (神)

(12)Buddha (仏)

(13)Ethics (倫理)

(14)Target (目標)

(15)World War II (第二次世界大戦)

(16)Pacific War (太平洋戦争)

(17)Militarism (軍国主義)

(18)Imperial Rescript on Education (教育勅語)

(19)Shushin/prewar moral education (修身)

(20)Paternalism (パターナリズム（押しつけ）)

(21)Masterful (偉そう)

(22)Strict (堅苦しいもの)

(23)Grumpy (気難しいもの)

(24)Old-fashioned (古くさいもの)

(25)Morality (道徳)

(26)Virtue education (徳育)

(27)Moral education (道徳教育)

(28)Virtue-ism (徳目主義)

(29)Character (人柄)

(30)Confucianism (儒教)

(31)Ideal (理想的)

(32)School (学校)

(33)School education (学校教育)

(34)Manly (男っぽい)

(35)Womanly (女っぽい)

(36)Compliance (コンプライアンス（法令遵守）)

(37)Power (力)

(38)Inborn (先天的（生まれつき）)

(39)Acquired (後天的)

(40)Others ( ) (その他（ ）)

(41)Nothing (連想するものはない)

10. Please select all the words below whose meanings you can hardly understand.

[* Subheadings were not displayed to the participants.]

Subheading 1, Western moral virtues

(1) Courage (勇気)

(2) Temperance (節制)

(3) Prudence (思慮深さ)

(4) Justice (正義)

(5) Piety (敬虔さ)

(6) Hope (希望)

(7) Love (愛)

(8) Generosity (気前よさ・物惜しみのなさ)

(9) Pride (志の高さ)

(10)Love for fame (名誉愛)

(11)Mild-temperedness (温和さ)

(12)Sociality (社交性)

(13)Honesty (正直さ)

(14)Wit (機知)

(15)Sense of shame (羞恥心)

(16)Legitimacy (合法)

(17)Equity (公平さ)

(18)Chastity (純潔・貞操)

(19)Abstinence (禁欲)

(20)Humility (謙遜)

(21)Effort (努力)

(22)Non-envy (嫉妬のなさ)

(23)Trust (信頼)

(24)Gratitude (感謝)

(25)Compassion (共感)

Subheading 2, Western epistemic virtues

(26)Memory (記憶力)

(27)Eyesight (視力)

(28)Hearing ability (聴力)

(29)Olfaction (嗅覚)

(30)Sense of taste (味覚)

(31)Tactility (触覚)

(32)Reasoning (推論能力)

(33)Autonomy (自律性)

(34)Understanding (理解力)

(35)Humility (謙遜)

(36)Non-discretion (非独断性)

(37)Non-gullibility (騙されにくさ)

(38)Non-egoism (非自己中心性)

(39)Non-self-complacency (自己満足で終わらないこと)

(40)Non-cruelness (冷徹でないこと)

(41)Responsibility (責任)

(42)Carefulness (慎重さ)

(43)Negligence (怠慢でないこと)

(44)Non-overconfidence (過信しないこと)

(45)Persistence (粘り強さ)

(46)Enthusiasm (熱意)

(47)Attentiveness (注意力)

(48)Sensitiveness (敏感さ)

(49)Self-discipline (自己鍛錬)

(50)Reflection (反省)

Subheading 3, Psychological Virtues

(51)Creativity (独創性)

(52)Curiosity (好奇心・興味)

(53)Open-mindedness (判断)

(54)Love of learning (向学心)

(55)Perspective (見通し)

(56)Bravery (勇敢)

(57)Persistence (勤勉)

(58)Integrity (誠実性)

(59)Vitality (熱意)

(60)Love (愛する力・愛される力)

(61)Kindness (親切)

(62)Social Intelligence (社会的知能)

(63)Leadership (リーダーシップ)

(64)Forgiveness and mercy (寛大)

(65)Humility and modesty (謙虚)

(66)Prudence (思慮深さ・慎重)

(67)Self-regulation (自己コントロール)

(68)Appreciation of beauty (審美心)

(69)Gratitude (感謝)

(70)Hope (希望・楽観性)

(71)Humor (ユーモア・遊戯心)

(72)Spirituality (精神性)

Subheading 4, Imperial Japanese moral and epistemic virtues

(73)Loyalty (忠君)

(74)Filial devotion (孝行)

(75)Fidelity (信義)

(76)Non-impoliteness (無礼でないこと)

(77)Studying (修学)

(78)Serving the public (公共奉仕)

(79)Patriotism (愛国心)

(80)Self-sacrifice (自己犠牲)

(81)Local patriotism (郷土愛)

Subheading 5, Confucian virtues

(82) Benevolence (仁)

(83) Loyalty (忠)

(84) Deference (恕)

(85) Trustworthiness (信)

(86) Ritual propriety (礼)

(87) Wisdom (知)

(88) Righteousness (義)

(89) Culture (文)

(90) Constant mean (中庸)

(91) Filial piety (孝)

(92) Fraternal respect (弟（悌）)

(93) Respectfulness (恭)

(94) Reverence (敬)

(95) Deference (譲)

(96) Humbleness (謙)

(97) Humility (孫（遜）)

(98) Earnest (勤)

(99) Uprightness (直)

(100) Stubbornness (諒)

(101) Genial (良)

(102) Carefulness (慎)

(103) Courage (勇)

(104) Elegancy (斯文)

(105) Fate (命)

(106) Arbiter of person (天)

(107) Way (道)

Subheading 6, Post-War Japanese moral and epistemic virtues

(108) Independence, autonomy, freedom and responsibility (自主、自律、自由と責任)

(109) Temperance (節度、節制)

(110) Ambition (向上心、個性の伸長)

(111) Hope, courage, self-denial, strong will (希望と勇気、克己と強い意志)

(112) Searching for truth, creation (真理の探究、創造)

(113) Compassion, gratitude (思いやり、感謝)

(114) Politeness (礼儀)

(115) Friendship, trust (友情、信頼)

(116) Mutual-understanding, broad-mindedness (相互理解、寛容)

(117) Spirit of law observance, spirit of public morality (遵法精神、公徳心)

(118) Fairness, equity, social justice (公正、公平、社会正義)

(119) Social participation, public spirit (社会参画、公共の精神)

(120) Laboring (勤労)

(121) Family love, repletion of home life (家族愛、家庭生活の充実)

(122) Better school life, repletion of group life (よりよい学校生活、集団生活の充実)

(123) Respect for local tradition and culture, love for hometown (郷土の伝統と文化の尊重、郷土を愛する態度)

(124) Respect for national tradition and culture, love for nation (我が国の伝統と文化の尊重、国を愛する態度)

(125) International understanding, international contribution (国際理解、国際貢献)

(126) Dignity of life (生命の尊さ)

(127) Nature conservation (自然愛護)

(128) Inspiration, awe-inspiring (感動、畏敬の念)

(129) Joy of living better (よりよく生きる喜び)

(130) No words whose meaning I do not understand

11. Please select up to five things you consider important for a person *qua* person, in the order of importance.

* If none of them are listed here, choose “N/A” in the “Most important” column.

(1) Courage (勇気)

(2) Temperance (節制)

(3) Prudence (思慮深さ)

(4) Justice (正義)

(5) Piety (敬虔さ)

(6) Hope (希望)

(7) Love (愛)

(8) Generosity (気前よさ・物惜しみのなさ)

(9) Pride (志の高さ)

(10) Love for fame (名誉愛)

(11) Mild-temperedness (温和さ)

(12) Sociality (社交性)

(13) Honesty (正直さ)

(14) Wit (機知)

(15) Sense of shame (羞恥心)

(16) Legitimacy (合法)

(17) Equity (公平さ)

(18) Chastity (純潔・貞操)

(19) Abstinence (禁欲)

(20) Humility (謙遜)

(21) Effort (努力)

(22) Non-envy (嫉妬のなさ)

(23) Trust (信頼)

(24) Gratitude (感謝)

(25) N/A

12. Please select up to five things you consider important for a person *qua* person, in the order of importance.

* If none of them are listed here, choose “N/A” in the “Most important” column.

(1) Memory (記憶力)

(2) Eyesight (視力)

(3) Hearing ability (聴力)

(4) Olfaction (嗅覚)

(5) Sense of taste (味覚)

(6) Tactility (触覚)

(7) Reasoning (推論能力)

(8) Autonomy (自律性)

(9) Understanding (理解力)

(10) Humility (謙遜)

(11) Non-discretion (非独断性)

(12) Non-gullibility (騙されにくさ)

(13) Non-egoism (非自己中心性)

(14) Non-self-complacency (自己満足で終わらないこと)

(15) Non-cruelness (冷徹でないこと)

(16) Responsibility (責任)

(17) Carefulness (慎重さ)

(18) Negligence (怠慢でないこと)

(19) Non-overconfidence (過信しないこと)

(20) Persistence (粘り強さ)

(21) Enthusiasm (熱意)

(22) Attentiveness (注意力)

(23) Sensitiveness (敏感さ)

(24) Self-discipline (自己鍛錬)

(25) Reflection (反省)

(26) N/A

13. Please select up to five things you consider important for a person *qua* person, in the order of importance.

* If none of them are listed here, choose “N/A” in the “Most important” column.

(1) Creativity (独創性)

(2) Curiosity (好奇心・興味)

(3) Open-mindedness (判断)

(4) Love of learning (向学心)

(5) Perspective (見通し)

(6) Bravery (勇敢)

(7) Persistence (勤勉)

(8) Integrity (誠実性)

(9) Vitality (熱意)

(10) Love (愛する力・愛される力)

(11) Kindness (親切)

(12) Social Intelligence (社会的知能)

(13) Leadership (リーダーシップ)

(14) Forgiveness and mercy (寛大)

(15) Humility and modesty (謙虚)

(16) Prudence (思慮深さ・慎重)

(17) Self-regulation (自己コントロール)

(18) Appreciation of beauty (審美心)

(19) Gratitude (感謝)

(20) Hope (希望・楽観性)

(21) Humor (ユーモア・遊戯心)

(22) Spirituality (精神性)

(23) N/A

14. Please select up to five things you consider important for a person *qua* person, in the order of importance.

* If none of them are listed here, choose “N/A” in the “Most important” column.

(1) Loyalty (忠君)

(2) Filial devotion (孝行)

(3) Fidelity (信義)

(4) Non-impoliteness (無礼でないこと)

(5) Studying (修学)

(6) Serving the public (公共奉仕)

(7) Patriotism (愛国心)

(8) Self-sacrifice (自己犠牲)

(9) Local patriotism (郷土愛)

(10) N/A

15. Please select up to five things you consider important for a person *qua* person, in the order of importance.

* If none of them are listed here, choose “N/A” in the “Most important” column.

(1) Benevolence (仁)

(2) Loyalty (忠)

(3) Deference (恕)

(4) Trustworthiness (信)

(5) Ritual propriety (礼)

(6) Wisdom (知)

(7) Righteousness (義)

(8) Culture (文)

(9) Constant mean (中庸)

(10) Filial piety (孝)

(11) Fraternal respect (弟（悌）)

(12) Respectful (恭)

(13) Reverence (敬)

(14) Deference (譲)

(15) Humbleness (謙)

(16) Humility (孫（遜）)

(17) Earnest (勤)

(18) Uprightness (直)

(19) Stubbornness (諒)

(20) Genial (良)

(21) Carefulness (慎)

(22) Courage (勇)

(23) Elegancy (斯文)

(24) Fate (命)

(25) Arbiter of person (天)

(26) Way (道)

(27) N/A

16. Please select up to five things you consider important for a person *qua* person, in the order of importance.

* If none of them are listed here, choose "N/A" in the "Most important" column.

(1) Independence, autonomy, freedom and responsibility (自主、自律、自由と責任)

(2) Temperance (節度、節制)

(3) Ambition (向上心、個性の伸長)

(4) Hope, courage, self-denial, strong will (希望と勇気、克己と強い意志)

(5) Searching for truth, creation (真理の探究、創造)

(6) Compassion, gratitude (思いやり、感謝)

(7) Politeness (礼儀)

(8) Friendship, trust (友情、信頼)

(9) Mutual-understanding, broad-mindedness (相互理解、寛容)

(10) Spirit of law observance, spirit of public morality (遵法精神、公徳心)

(11) Fairness, equity, social justice (公正、公平、社会正義)

(12) Social participation, public spirit (社会参画、公共の精神)

(13) Laboring (勤労)

(14) Family love, repletion of home life (家族愛、家庭生活の充実)

(15) Better school life, repletion of group life (よりよい学校生活、集団生活の充実)

(16) Respect for local tradition and culture, love for hometown (郷土の伝統と文化の尊重、郷土を愛する態度)

(17) Respect for national tradition and culture, love for nation (我が国の伝統と文化の尊重、国を愛する態度)

(18) International understanding, international contribution (国際理解、国際貢献)

(19) Dignity of life (生命の尊さ)

(20) Nature conservation (自然愛護)

(21) Inspiration, awe-inspiring (感動、畏敬の念)

(22) Joy of living better (よりよく生きる喜び)

(23) N/A

17. (Free answer) Please feel free to write any comments or opinions you may have about this survey. (Optional answer)
