# Supplementary material for: The consciousness of virtue: uncovering the gaps between educational specialists and the general public in their understanding of virtue in Japan
Source: Front Psychol. 2024 Feb 15;14:1171247. doi: 10.3389/fpsyg.2023.1171247 (PMC10902463; doi:10.3389/fpsyg.2023.1171247)
Supplement: Supplementary file 2 [file Table_1.pdf]

Supplementary 2. Table: Virtues considered important

This table is the complete version of Table 5. For each category, the respondents were asked to select up to the fifth most important virtue. This table deals with the first of these. For comparisons between the GP and ES and also PR and npr-ES, after Fisher's exact probability test, multiplicity was adjusted by Holm's method and p-values were obtained. When the ratio was significantly greater for ES than for the GP and for PR than for npr-ES, * denotes p < 0.05, and ** denotes p < 0.01. When the ratio was significantly smaller for ES than for the GP and for PR than for npr-ES, † indicates p < 0.05, and †† indicates p < 0.01. Respondents were asked to select up to 3rd place, but only 1st place was used in the analysis. The reason was that the strength of preference between 2nd place and 3rd place was unknown, and we thought that only using 1st place for analysis would better reflect the subject's preference. GP: general public, ES: educational specialists, PS: professional researchers, npr-ES: non-professional educational specialists.

	virtue	GP		ES														
Category				Total		Percentage difference	Effect size	p-value with Fisher's exact probability test	p-value adjusted for multiplicity by Holm's method		PR		npr-ES		Percentage difference	Effect size	p-value with Fisher's exact probability test	p-value adjusted for multiplicity by Holm's method
1	Courage (勇気)	12	1.2%	3	1.1%	0.1%	0.01	1.00	1.00		2	1.0%	1	1.3%	-0.3%	-0.02	1.00	1.00
	Temperance (節制)	2	0.2%	0	0.0%	0.2%	0.09	1.00	1.00		0	0.0%	0	0.0%	0.0%	0.00	—	—
	Prudence (思慮深さ)	47	4.7%	31	11.4%	-6.7%	-0.25	0.00	0.00	**	21	10.8%	10	13.0%	-2.2%	-0.07	0.67	1.00
	Justice (正義)	23	2.3%	9	3.3%	-1.0%	-0.06	0.38	1.00		7	3.6%	2	2.6%	1.0%	0.06	1.00	1.00
	Piety (敬虔さ)	4	0.4%	4	1.5%	-1.1%	-0.12	0.07	1.00		4	2.1%	0	0.0%	2.1%	0.29	0.58	1.00
	Hope (希望)	20	2.0%	19	7.0%	-5.0%	-0.25	0.00	0.00	**	15	7.7%	4	5.2%	2.5%	0.10	0.60	1.00
	Love (愛)	143	14.3%	49	18.1%	-3.7%	-0.10	0.13	1.00		33	17.0%	16	20.8%	-3.8%	-0.10	0.49	1.00
	Generosity (気前よさ・物惜しみのなさ)	3	0.3%	3	1.1%	-0.8%	-0.10	0.12	1.00		2	1.0%	1	1.3%	-0.3%	-0.02	1.00	1.00
	Pride (志の高さ)	16	1.6%	7	2.6%	-1.0%	-0.07	0.30	1.00		5	2.6%	2	2.6%	0.0%	0.00	1.00	1.00

	Love for fame (名誉愛)	0	0.0%	0	0.0%	0.0%	0.00	—	—		0	0.0%	0	0.0%	0.0%	0.00	—	—
	Mild-temperedness (温和さ)	33	3.3%	6	2.2%	1.1%	0.07	0.43	1.00		3	1.5%	3	3.9%	-2.3%	-0.15	0.36	1.00
	Sociality (社交性)	19	1.9%	3	1.1%	0.8%	0.07	0.60	1.00		1	0.5%	2	2.6%	-2.1%	-0.18	0.20	1.00
	Honesty (正直さ)	136	13.6%	27	10.0%	3.7%	0.11	0.12	1.00		21	10.8%	6	7.8%	3.0%	0.10	0.51	1.00
	Wit (機知)	0	0.0%	4	1.5%	-1.5%	-0.24	0.00	0.04	*	4	2.1%	0	0.0%	2.1%	0.29	0.58	1.00
	Sense of shame (羞恥心)	2	0.2%	2	0.7%	-0.5%	-0.08	0.20	1.00		2	1.0%	0	0.0%	1.0%	0.20	1.00	1.00
	Legitimacy (合法)	9	0.9%	0	0.0%	0.9%	0.19	0.22	1.00		0	0.0%	0	0.0%	0.0%	0.00	—	—
	Equity (公平さ)	35	3.5%	9	3.3%	0.2%	0.01	1.00	1.00		9	4.6%	0	0.0%	4.6%	0.43	0.06	1.00
	Chastity (純潔・貞操)	2	0.2%	0	0.0%	0.2%	0.09	1.00	1.00		0	0.0%	0	0.0%	0.0%	0.00	—	—
	Abstinence (禁欲)	1	0.1%	0	0.0%	0.1%	0.06	1.00	1.00		0	0.0%	0	0.0%	0.0%	0.00	—	—
	Humility (謙遜)	28	2.8%	1	0.4%	2.4%	0.22	0.01	0.23		1	0.5%	0	0.0%	0.5%	0.14	1.00	1.00
	Effort (努力)	28	2.8%	4	1.5%	1.3%	0.09	0.28	1.00		4	2.1%	0	0.0%	2.1%	0.29	0.58	1.00
	Non-envy (嫉妬のなさ)	4	0.4%	2	0.7%	-0.3%	-0.05	0.61	1.00		1	0.5%	1	1.3%	-0.8%	-0.08	0.49	1.00
	Trust (信頼)	95	9.5%	20	7.4%	2.1%	0.08	0.34	1.00		11	5.7%	9	11.7%	-6.0%	-0.22	0.12	1.00
	Gratitude (感謝)	264	26.5%	25	9.2%	17.3%	0.46	0.00	0.00	††	12	6.2%	13	16.9%	-10.7%	-0.34	0.01	0.20
	Compassion (共感)	7	0.7%	20	7.4%	-6.7%	-0.38	0.00	0.00	**	15	7.7%	5	6.5%	1.2%	0.05	1.00	1.00
	Not applicable	64	6.4%	23	8.5%	-2.1%	-0.08	0.23	1.00		21	10.8%	2	2.6%	8.2%	0.35	0.03	0.59
2	Memory (記憶力)	5	0.5%	3	1.1%	-0.6%	-0.07	0.38	1.00		3	1.5%	0	0.0%	1.5%	0.25	0.56	1.00
	Eyesight (視力)	22	2.2%	0	0.0%	2.2%	0.30	0.01	0.15		0	0.0%	0	0.0%	0.0%	0.00	—	—
	Hearing ability (聴力)	10	1.0%	0	0.0%	1.0%	0.20	0.13	1.00		0	0.0%	0	0.0%	0.0%	0.00	—	—
	Olfaction (嗅覚)	0	0.0%	0	0.0%	0.0%	0.00	—	—		0	0.0%	0	0.0%	0.0%	0.00	—	—
	Sense of taste (味覚)	6	0.6%	1	0.4%	0.2%	0.03	1.00	1.00		0	0.0%	1	1.3%	-1.3%	-0.23	0.28	1.00
	Tactility (触覚)	0	0.0%	1	0.4%	-0.4%	-0.12	0.21	1.00		1	0.5%	0	0.0%	0.5%	0.14	1.00	1.00

Reasoning (推論能力)	6	0.6%	7	2.6%	-2.0%	-0.17	0.01	0.18		6	3.1%	1	1.3%	1.8%	0.13	0.68	1.00
Autonomy (自律性)	92	9.2%	69	25.5%	-16.2%	-0.44	0.00	0.00	**	43	22.2%	26	33.8%	-11.6%	-0.26	0.06	1.00
Understanding (理解力)	78	7.8%	18	6.6%	1.2%	0.05	0.60	1.00		12	6.2%	6	7.8%	-1.6%	-0.06	0.60	1.00
Humility (謙遜)	73	7.3%	8	3.0%	4.4%	0.20	0.01	0.15		6	3.1%	2	2.6%	0.5%	0.03	1.00	1.00
Non-discretion (非独断性)	5	0.5%	10	3.7%	-3.2%	-0.24	0.00	0.00	**	7	3.6%	3	3.9%	-0.3%	-0.02	1.00	1.00
Non-gullibility (騙されにくさ)	7	0.7%	2	0.7%	0.0%	0.00	1.00	1.00		1	0.5%	1	1.3%	-0.8%	-0.08	0.49	1.00
Non-egoism (非自己中心性)	45	4.5%	18	6.6%	-2.1%	-0.09	0.16	1.00		14	7.2%	4	5.2%	2.0%	0.08	0.79	1.00
Non-self-complacency (自己満足で終わらないこと)	30	3.0%	5	1.8%	1.2%	0.08	0.40	1.00		4	2.1%	1	1.3%	0.8%	0.06	1.00	1.00
Non-cruelness (冷徹でないこと)	18	1.8%	7	2.6%	-0.8%	-0.05	0.46	1.00		6	3.1%	1	1.3%	1.8%	0.13	0.68	1.00
Responsibility (責任)	201	20.2%	27	10.0%	10.2%	0.29	0.00	0.00	††	15	7.7%	12	15.6%	-7.9%	-0.25	0.07	1.00
Carefulness (慎重さ)	28	2.8%	8	3.0%	-0.1%	-0.01	0.84	1.00		5	2.6%	3	3.9%	-1.3%	-0.07	0.69	1.00
Negligence (怠慢でないこと)	42	4.2%	2	0.7%	3.5%	0.24	0.00	0.09		1	0.5%	1	1.3%	-0.8%	-0.08	0.49	1.00
Non-overconfidence (過信しないこと)	55	5.5%	10	3.7%	1.8%	0.09	0.28	1.00		8	4.1%	2	2.6%	1.5%	0.09	0.73	1.00
Persistence (粘り強さ)	25	2.5%	4	1.5%	1.0%	0.07	0.49	1.00		2	1.0%	2	2.6%	-1.6%	-0.12	0.32	1.00
Enthusiasm (熱意)	46	4.6%	5	1.8%	2.8%	0.16	0.04	0.63		2	1.0%	3	3.9%	-2.9%	-0.19	0.14	1.00

	Attentiveness (注意力)	13	1.3%	2	0.7%	0.6%	0.06	0.75	1.00		1	0.5%	1	1.3%	-0.8%	-0.08	0.49	1.00
	Sensitiveness (敏感さ)	4	0.4%	3	1.1%	-0.7%	-0.08	0.17	1.00		3	1.5%	0	0.0%	1.5%	0.25	0.56	1.00
	Self-discipline (自己鍛錬)	43	4.3%	9	3.3%	1.0%	0.05	0.60	1.00		8	4.1%	1	1.3%	2.8%	0.18	0.45	1.00
	Reflection (反省)	34	3.4%	23	8.5%	-5.1%	-0.22	0.00	0.02	*	20	10.3%	3	3.9%	6.4%	0.26	0.10	1.00
	Not applicable	109	10.9%	29	10.7%	0.2%	0.01	1.00	1.00		26	13.4%	3	3.9%	9.5%	0.35	0.03	0.63
3	Creativity (独創性)	6	0.6%	4	1.5%	-0.9%	-0.09	0.23	1.00		2	1.0%	2	2.6%	-1.6%	-0.12	0.32	1.00
	Curiosity (好奇心・興味)	30	3.0%	12	4.4%	-1.4%	-0.08	0.25	1.00		9	4.6%	3	3.9%	0.7%	0.04	1.00	1.00
	Open-mindedness (判断)	26	2.6%	4	1.5%	1.1%	0.08	0.37	1.00		3	1.5%	1	1.3%	0.2%	0.02	1.00	1.00
	Love of learning (向学心)	15	1.5%	4	1.5%	0.0%	0.00	1.00	1.00		4	2.1%	0	0.0%	2.1%	0.29	0.58	1.00
	Perspective (見通し)	3	0.3%	1	0.4%	-0.1%	-0.01	1.00	1.00		0	0.0%	1	1.3%	-1.3%	-0.23	0.28	1.00
	Bravery (勇敢)	4	0.4%	0	0.0%	0.4%	0.13	0.58	1.00		0	0.0%	0	0.0%	0.0%	0.00	—	—
	Persistence (勤勉)	18	1.8%	3	1.1%	0.7%	0.06	0.59	1.00		2	1.0%	1	1.3%	-0.3%	-0.02	1.00	1.00
	Integrity (誠実性)	123	12.3%	68	25.1%	-12.8%	-0.33	0.00	0.00	**	46	23.7%	22	28.6%	-4.9%	-0.11	0.44	1.00
	Vitality (熱意)	15	1.5%	2	0.7%	0.8%	0.07	0.55	1.00		2	1.0%	0	0.0%	1.0%	0.20	1.00	1.00
	Love (愛する力・愛される力)	96	9.6%	24	8.9%	0.8%	0.03	0.81	1.00		17	8.8%	7	9.1%	-0.3%	-0.01	1.00	1.00
	Kindness (親切)	56	5.6%	8	3.0%	2.7%	0.13	0.09	1.00		7	3.6%	1	1.3%	2.3%	0.15	0.45	1.00
	Social Intelligence (社会的知能)	21	2.1%	4	1.5%	0.6%	0.05	0.63	1.00		3	1.5%	1	1.3%	0.2%	0.02	1.00	1.00
	Leadership (リーダーシップ)	5	0.5%	0	0.0%	0.5%	0.14	0.59	1.00		0	0.0%	0	0.0%	0.0%	0.00	—	—
	Forgiveness and mercy (寛大)	28	2.8%	12	4.4%	-1.6%	-0.09	0.17	1.00		10	5.2%	2	2.6%	2.6%	0.13	0.52	1.00

	Humility and modesty (謙虚)	48	4.8%	4	1.5%	3.3%	0.20	0.01	0.26		4	2.1%	0	0.0%	2.1%	0.29	0.58	1.00	
	Prudence (思慮深さ・慎重)	34	3.4%	25	9.2%	-5.8%	-0.25	0.00	0.00	**	20	10.3%	5	6.5%	3.8%	0.14	0.48	1.00	
	Self-regulation (自己コントロール)	54	5.4%	9	3.3%	2.1%	0.10	0.21	1.00		3	1.5%	6	7.8%	-6.2%	-0.32	0.02	0.37	
	Appreciation of beauty (審美心)	4	0.4%	3	1.1%	-0.7%	-0.08	0.17	1.00		3	1.5%	0	0.0%	1.5%	0.25	0.56	1.00	
	Gratitude (感謝)	278	27.9%	26	9.6%	18.3%	0.48	0.00	0.00	††	13	6.7%	13	16.9%	-10.2%	-0.32	0.02	0.40	
	Hope (希望・楽観性)	21	2.1%	18	6.6%	-4.5%	-0.23	0.00	0.01	**	14	7.2%	4	5.2%	2.0%	0.08	0.79	1.00	
	Humor (ユーモア・遊戯心)	12	1.2%	6	2.2%	-1.0%	-0.08	0.24	1.00		5	2.6%	1	1.3%	1.3%	0.09	1.00	1.00	
	Spirituality (精神性)	20	2.0%	14	5.2%	-3.2%	-0.17	0.01	0.17		9	4.6%	5	6.5%	-1.9%	-0.08	0.55	1.00	
	Not applicable	80	8.0%	20	7.4%	0.6%	0.02	0.80	1.00		18	9.3%	2	2.6%	6.7%	0.30	0.07	1.00	
4	Loyalty (忠君)	6	0.6%	2	0.7%	-0.1%	-0.02	0.68	1.00		2	1.0%	0	0.0%	1.0%	0.20	1.00	1.00	
	Filial devotion (孝行)	148	14.8%	18	6.6%	8.2%	0.27	0.00	0.00	††	6	3.1%	12	15.6%	-12.5%	-0.46	0.00	0.01	††
	Fidelity (信義)	202	20.3%	73	26.9%	-6.8%	-0.16	0.02	0.12		52	26.8%	21	27.3%	-0.3%	-0.01	1.00	1.00	
	Non-impoliteness (無礼でないこと)	277	27.8%	47	17.3%	10.4%	0.25	0.00	0.00	††	35	18.0%	12	15.6%	2.6%	0.07	0.72	1.00	
	Studying (修学)	47	4.7%	32	11.8%	-7.1%	-0.27	0.00	0.00	**	23	11.9%	9	11.7%	0.2%	0.01	1.00	1.00	
	Serving the public (公共奉仕)	29	2.9%	20	7.4%	-4.5%	-0.21	0.00	0.01	*	12	6.2%	8	10.4%	-4.2%	-0.15	0.30	1.00	
	Patriotism (愛国心)	27	2.7%	2	0.7%	2.0%	0.16	0.06	0.32		2	1.0%	0	0.0%	1.0%	0.20	1.00	1.00	
	Self-sacrifice (自己犠牲)	22	2.2%	5	1.8%	0.4%	0.03	1.00	1.00		2	1.0%	3	3.9%	-2.9%	-0.19	0.14	1.00	
	Local patriotism (郷土愛)	20	2.0%	4	1.5%	0.5%	0.04	0.80	1.00		4	2.1%	0	0.0%	2.1%	0.29	0.58	1.00	
	Not applicable	219	22.0%	67	24.7%	-2.8%	-0.07	0.33	1.00		55	28.4%	12	15.6%	12.9%	0.31	0.03	0.26	

5	Benevolence (仁)	37	3.7%	43	15.9%	-12.2%	-0.43	0.00	0.00	**	28	14.4%	15	19.5%	-5.0%	-0.13	0.36	1.00
	Loyalty (忠)	7	0.7%	0	0.0%	0.7%	0.17	0.36	1.00		0	0.0%	0	0.0%	0.0%	0.00	—	—
	Deference (恕)	3	0.3%	13	4.8%	-4.5%	-0.33	0.00	0.00	**	10	5.2%	3	3.9%	1.3%	0.06	0.76	1.00
	Trustworthiness (信)	50	5.0%	13	4.8%	0.2%	0.01	1.00	1.00		10	5.2%	3	3.9%	1.3%	0.06	0.76	1.00
	Ritual propriety (礼)	122	12.2%	13	4.8%	7.5%	0.27	0.00	0.01	††	6	3.1%	7	9.1%	-6.0%	-0.26	0.06	1.00
	Wisdom (知)	26	2.6%	20	7.4%	-4.8%	-0.23	0.00	0.01	*	16	8.2%	4	5.2%	3.1%	0.12	0.45	1.00
	Righteousness (義)	41	4.1%	14	5.2%	-1.1%	-0.05	0.50	1.00		9	4.6%	5	6.5%	-1.8%	-0.08	0.55	1.00
	Culture (文)	1	0.1%	2	0.7%	-0.6%	-0.11	0.12	1.00		2	1.0%	0	0.0%	1.0%	0.20	1.00	1.00
	Constant mean (中庸)	11	1.1%	35	12.9%	-11.9%	-0.53	0.00	0.00	**	30	15.5%	5	6.5%	9.1%	0.30	0.05	1.00
	Filial piety (孝)	7	0.7%	3	1.1%	-0.4%	-0.04	0.45	1.00		1	0.5%	2	2.6%	-2.1%	-0.18	0.20	1.00
	Fraternal respect (弟（悌）)	2	0.2%	0	0.0%	0.2%	0.09	1.00	1.00		0	0.0%	0	0.0%	0.0%	0.00	—	—
	Respectful (恭)	1	0.1%	0	0.0%	0.1%	0.06	1.00	1.00		0	0.0%	0	0.0%	0.0%	0.00	—	—
	Reverence (敬)	46	4.6%	7	2.6%	2.0%	0.11	0.17	1.00		5	2.6%	2	2.6%	0.0%	0.00	1.00	1.00
	Deference (譲)	4	0.4%	1	0.4%	0.0%	0.01	1.00	1.00		0	0.0%	1	1.3%	-1.3%	-0.23	0.29	1.00
	Humbleness (謙)	21	2.1%	4	1.5%	0.6%	0.05	0.63	1.00		3	1.5%	1	1.3%	0.3%	0.02	1.00	1.00
	Humility (孫（遜）)	2	0.2%	1	0.4%	-0.2%	-0.03	0.51	1.00		1	0.5%	0	0.0%	0.5%	0.14	1.00	1.00
	Earnest (勤)	14	1.4%	1	0.4%	1.0%	0.12	0.22	1.00		1	0.5%	0	0.0%	0.5%	0.14	1.00	1.00
	Uprightness (直)	18	1.8%	3	1.1%	0.7%	0.06	0.59	1.00		1	0.5%	2	2.6%	-2.1%	-0.18	0.20	1.00
	Stubbornness (諒)	1	0.1%	1	0.4%	-0.3%	-0.06	0.38	1.00		1	0.5%	0	0.0%	0.5%	0.14	1.00	1.00
	Genial (良)	30	3.0%	2	0.7%	2.3%	0.18	0.03	0.59		1	0.5%	1	1.3%	-0.8%	-0.08	0.49	1.00
	Carefulness (慎)	4	0.4%	1	0.4%	0.0%	0.01	1.00	1.00		1	0.5%	0	0.0%	0.5%	0.14	1.00	1.00
	Courage (勇)	7	0.7%	4	1.5%	-0.8%	-0.08	0.26	1.00		4	2.1%	0	0.0%	2.1%	0.29	0.58	1.00
	Elegancy (斯文)	1	0.1%	0	0.0%	0.1%	0.06	1.00	1.00		0	0.0%	0	0.0%	0.0%	0.00	—	—
	Fate (命)	311	31.2%	30	11.1%	20.2%	0.51	0.00	0.00	††	19	9.8%	11	14.3%	-4.4%	-0.14	0.29	1.00
	Arbiter of person (天)	2	0.2%	3	1.1%	-0.9%	-0.12	0.07	1.00		3	1.5%	0	0.0%	1.6%	0.25	0.56	1.00
	Way (道)	22	2.2%	14	5.2%	-3.0%	-0.16	0.01	0.28		10	5.2%	4	5.2%	0.0%	0.00	1.00	1.00

	Not applicable	201	20.2%	42	15.5%	4.7%	0.12	0.10	1.00		31	16.0%	11	14.3%	1.8%	0.05	0.85	1.00
6	Independence, autonomy, freedom and responsibility (自主、自律、自由と責任)	32	3.2%	31	11.4%	-8.2%	-0.33	0.00	0.00	**	26	13.4%	5	6.5%	6.9%	0.23	0.14	1.00
	Temperance (節度、節制)	9	0.9%	0	0.0%	0.9%	0.19	0.22	1.00		0	0.0%	0	0.0%	0.0%	0.00	—	—
	Ambition (向上心、個性の伸長)	23	2.3%	2	0.7%	1.6%	0.13	0.14	1.00		1	0.5%	1	1.3%	-0.8%	-0.08	0.49	1.00
	Hope, courage, self-denial, strong will (希望と勇気、克己と強い意志)	20	2.0%	5	1.8%	0.2%	0.01	1.00	1.00		4	2.1%	1	1.3%	0.8%	0.06	1.00	1.00
	Searching for truth, creation (真理の探究、創造)	4	0.4%	17	6.3%	-5.9%	-0.38	0.00	0.00	**	14	7.2%	3	3.9%	3.3%	0.15	0.41	1.00
	Compassion, gratitude (思いやり、感謝)	310	31.1%	40	14.8%	16.3%	0.39	0.00	0.00	††	21	10.8%	19	24.7%	-13.9%	-0.37	0.01	0.14
	Politeness (礼儀)	88	8.8%	2	0.7%	8.1%	0.43	0.00	0.00	††	2	1.0%	0	0.0%	1.0%	0.20	1.00	1.00
	Friendship, trust (友情、信頼)	29	2.9%	4	1.5%	1.4%	0.10	0.28	1.00		3	1.5%	1	1.3%	0.2%	0.02	1.00	1.00
	Mutual-understanding, broad-mindedness (相互理解、寛容)	28	2.8%	31	11.4%	-8.6%	-0.35	0.00	0.00	**	24	12.4%	7	9.1%	3.3%	0.11	0.53	1.00
	Spirit of law observance, spirit of public morality (遵法精神、公徳心)	12	1.2%	2	0.7%	0.5%	0.05	0.75	1.00		0	0.0%	2	2.6%	-2.6%	-0.32	0.08	1.00

Fairness, equity, social justice (公正、公平、社会正義)	28	2.8%	32	11.8%	-9.0%	-0.36	0.00	0.00	**	27	13.9%	5	6.5%	7.4%	0.25	0.10	1.00	
Social participation, public spirit (社会参画、公共の精神)	3	0.3%	1	0.4%	-0.1%	-0.01	1.00	1.00		1	0.5%	0	0.0%	0.5%	0.14	1.00	1.00	
Laboring (勤労)	12	1.2%	1	0.4%	0.8%	0.10	0.32	1.00		0	0.0%	1	1.3%	-1.3%	-0.23	0.28	1.00	
Family love, repletion of home life (家族愛、家庭生活の充実)	67	6.7%	3	1.1%	5.6%	0.31	0.00	0.00	††	2	1.0%	1	1.3%	-0.3%	-0.02	1.00	1.00	
Better school life, repletion of group life (よりよい学校生活、集団生活の充実)	4	0.4%	1	0.4%	0.0%	0.01	1.00	1.00		0	0.0%	1	1.3%	-1.3%	-0.23	0.28	1.00	
Respect for local tradition and culture, love for hometown (郷土の伝統と文化の尊重、郷土を愛する態度)	6	0.6%	1	0.4%	0.2%	0.03	1.00	1.00		1	0.5%	0	0.0%	0.5%	0.14	1.00	1.00	
Respect for national tradition and culture, love for nation (我が国の伝統と文化の尊重、国を愛する態度)	4	0.4%	2	0.7%	-0.3%	-0.05	0.61	1.00		1	0.5%	1	1.3%	-0.8%	-0.08	0.49	1.00	

International understanding, international contribution (国際理解、国際貢献)	1	0.1%	0	0.0%	0.1%	0.06	1.00	1.00		0	0.0%	0	0.0%	0.0%	0.00	—	—
Dignity of life (生命の尊さ)	180	18.1%	39	14.4%	3.7%	0.10	0.17	1.00		26	13.4%	13	16.9%	-3.5%	-0.10	0.45	1.00
Nature conservation (自然愛護)	4	0.4%	0	0.0%	0.4%	0.13	0.58	1.00		0	0.0%	0	0.0%	0.0%	0.00	—	—
Inspiration, awe-inspiring (感動、畏敬の念)	10	1.0%	6	2.2%	-1.2%	-0.10	0.13	1.00		5	2.6%	1	1.3%	1.3%	0.09	1.00	1.00
Joy of living better (よりよく生きる喜び)	37	3.7%	29	10.7%	-7.0%	-0.28	0.00	0.00	**	17	8.8%	12	15.6%	-6.8%	-0.21	0.13	1.00
Not applicable	86	8.6%	22	8.1%	0.5%	0.02	0.90	1.00		19	9.8%	3	3.9%	5.9%	0.24	0.14	1.00
